# Comparison Study of Nontreated and Fermented Wheat Varieties ‘Ada’, ‘Sarta’, and New Breed Blue and Purple Wheat Lines Wholemeal Flour

**DOI:** 10.3390/biology11070966

**Published:** 2022-06-27

**Authors:** Elena Bartkiene, Vytaute Starkute, Egle Zokaityte, Dovile Klupsaite, Ernestas Mockus, Vadims Bartkevics, Anastasija Borisova, Romas Gruzauskas, Žilvinas Liatukas, Vytautas Ruzgas

**Affiliations:** 1 Institute of Animal Rearing Technologies, Lithuanian University of Health Sciences, Tilzes Str. 18, LT-47181 Kaunas, Lithuania; vytaute.starkute@lsmuni.lt (V.S.); egle.zokaityte@lsmuni.lt (E.Z.); dovile.klupsaite@lsmuni.lt (D.K.); ernestas.mockus@lsmuni.lt (E.M.); 2 Department of Food Safety and Quality, Lithuanian University of Health Sciences, Tilzes G. 18, LT-47181 Kaunas, Lithuania; 3 Institute of Food Safety, Animal Health and Environment BIOR, Lejupes Iela 3, LV-1076 Riga, Latvia; vadims.bartkevics@bior.lv (V.B.); anastasija.borisova@bior.lv (A.B.); 4 Department of Food Science and Technology, Kaunas University of Technology, Radvilenu Rd. 19, LT-50254 Kaunas, Lithuania; romas.gruzauskas@ktu.lt; 5 Institute of Agriculture, Lithuanian Research Centre for Agriculture and Forestry, Instituto al. 1, Akademija, LT-58344 Kedainiai, Lithuania; zilvinas.liatukas@lammc.lt (Ž.L.); vytautas.ruzgas@lammc.lt (V.R.)

**Keywords:** wheat, fermentation, biogenic amines, volatile compounds, mycotoxins

## Abstract

**Simple Summary:**

Nowadays, consumers are looking for higher functional value products that can meet the nutritional requirements of functional compounds. Wheat is the most important crop in the world, and to improve its functional properties, colored wheat has become a very attractive option. However, most of the valuable compounds of the wheat cereal (including colored) are located in the outer layer of the cereal. From this point of view, application of non-refined (wholemeal) flour became very important. The aim of this study was to analyze and compare physico-chemical and microbiological parameters of the nontreated ‘Ada’, ‘Sarta’, and new breed blue and purple wholemeal wheat lines with those fermented with lactic acid bacteria (LAB) strains (*Pediococcus acidilactici*, *Liquorilactobacillus uvarum*, *Lactiplantibacillus plantarum*). It was found that all of the used LAB strains are suitable for the tested wheat wholemeal fermentation. However, most of the analyzed parameters were influenced by the wheat variety, the type of LAB used for fermentation, and their interaction. Despite the fact that fermented wheat wholemeal showed a large number of viable LAB, good acidification rates, and a more diverse volatile compound profile. further studies are needed to indicate the technological parameters that lead to the lowest BA formation and mycotoxin degradation.

**Abstract:**

The aim of this study was to analyze and compare the acidity, microbiological, and chromaticity parameters; fatty acid (FA) and volatile compound (VC) profiles; and biogenic amine (BA), macro- and microelement, and mycotoxin concentrations in nontreated ‘Ada’, ‘Sarta’, and new breed blue (DS8472-5) and purple (DS8526-2) wheat lines wholemeal (WW) with those fermented with lactic acid bacteria (LAB) possessing antimicrobial/antifungal properties, isolated from spontaneous sourdough: *Pediococcus acidilactici*-LUHS29, *Liquorilactobacillus uvarum*-LUHS245, *Lactiplantibacillus plantarum*-LUHS122). All the fermented WW showed >8.0 log_10_ CFU/g of LAB count, and the type of LAB was a significant factor in the WW acidity parameters. Phenylethylamine was the predominant BA in WW, and the wheat variety (WV), the type of LAB, and their interaction were significant factors on the BA formation. Despite the fact that some differences in trace element concentrations in WW were obtained, in most of the cases fermentation was not a significant factor in their content. The main FAs in WW were palmitic acid, all-*cis,trans*-octadecenoic acid, and linoleic acid. Fermented WW showed a more diverse VC profile; however, the influence of fermentation on deoxynivalenol in WW was varied. Finally, further studies are needed to indicate the technological parameters that would be the most effective for each WV, including the lowest BA formation and mycotoxin degradation.

## 1. Introduction

Wheat is the most important food and feed crop in the world [1]. It provides many beneficial nutrients (vitamins B and E; minerals iron and zinc; various phytochemicals, etc.) [2]. The grains of traditional wheat (*T. aestivum*) varieties are amber in color; however, nowadays, colored wheat grain has become a very attractive option because of the higher content of anthocyanins and other phytochemicals [3,4], which provide numerous health benefits [3,5,6]. Also, the antimicrobial activity of these valuable compounds against different Gram-positive and Gram-negative human pathogens could be very promising [7,8]. However, on the other hand, antimicrobial compounds could influence the fermentation process, which is the main technological step in sourdough bread preparation. For this reason, in this study, in addition to the traditional wheat variety (‘Ada’), the waxy wheat variety (‘Sarta’), and new breed lines of the colored wheat (blue wheat DS8472-5 and purple wheat DS8526-2) for wheat wholemeal (WW) sourdough preparation were tested.

Despite the fact that larger amounts of whole grain products are recommended in the human diet [9,10,11,12], there are many challenges associated with the technological and safety characteristics of such types of products. To improve the technological and safety characteristics of WW, fermentation with selected lactic acid bacteria (LAB) strains could be suggested. Also, our previous studies showed that a combination of extrusion and fermentation technologies for wheat bran pre-treatment could be a very attractive solution for the reduction of biogenic amines (BAs) and mycotoxins [13,14].

Overall, sourdough is a mixture consisting mainly of cereal grain flour and water fermented by a heterogeneous microbiota, with predominantly LAB and yeast strains [15,16]. However, characteristics of spontaneous sourdough are dependent on many factors (contamination of flour, environment, etc.). In addition, when WW is used, higher contamination of the outer layer should be taken into consideration. For this reason, the use of selected LAB starter cultures, which possess antifungal and antimicrobial properties and/or are capable of degrading mycotoxins, could be very promising. Also, during the sourdough fermentation process, enzymatic and microbial changes lead to the formation of various volatile compounds (VCs) that improve the overall acceptability of the cereal products [17,18]. However, studies regarding the sourdough VCs are scarce [19]. The acidification properties, microbiological characteristics, and VC production are interesting properties of the sourdough, and the selection of LAB starters has become important to improve the technological properties of cereal products.

Factors affecting sourdough fermentation include microbial diversity, fermentation conditions, and flour composition [20]. In this study, four wheat varieties (‘Ada’, ‘Sarta’, new breed lines of the colored wheat known as blue wheat DS8472-5 and purple wheat DS8526-2), and three LAB strains (*Pediococcus acidilactici*, *Liquorilactobacillus uvarum*, *Lactiplantibacillus plantarum*) possessing antimicrobial properties were tested for WW sourdough preparation. The antimicrobial and antifungal properties as well as the metabolic capacity to ferment a large number of carbohydrate sources were taken into consideration in the selection of LAB strains for sourdough preparation [21].

Despite the fact that fermented wheat products are associated with many health benefits [22,23,24,25,26], it should be pointed out that lactofermentation is also associated with the formation of biogenic amines (BAs), which are toxic nitrogenous bases formed by the decarboxylation of amino acids. The tolerance ranges of some BAs have been suggested: histamine, 50–100 mg/kg; tyramine, 100–800 mg/kg; and 2-phenylethylamine, 30 mg/kg [27,28]. Presently, for putrescine and cadaverine, no toxic concentrations were available [29]. However, spermine and spermidine increases the toxicity of histamine and tyramine [30]. For this reason, analysis of the BAs of the prepared sourdoughs in this study was included.

In addition, the fatty acid (FA) profile of the prepared sourdoughs was analyzed. It was reported that hydroxy FAs produced by sourdough LAB exhibit antifungal activity [31], which is related to the increase in the fungal membrane permeability [31]. Also, when sourdough is prepared from cereal bran and/or WW, both the antifungal and mycotoxin degradation properties of the used LAB starters for sourdough preparation are very important. Despite the fact that aflatoxins B1, B2, G1, and G2 are the most important known carcinogenic mycotoxins [16], in addition to regulated mycotoxins, emerging and masking mycotoxins should be controlled. Emerging toxins are a group of mycotoxins for which no regulations exist at the present [32]. The clinical observations in animals in some cases of mycotoxicosis did not correlate with the low mycotoxin content determined in the corresponding feed [33]. The unexpectedly high toxicity has been attributed to undetected conjugated forms of mycotoxins that were possibly hydrolyzed in vivo [32]. Our previous studies showed that cereal pre-treatment by using fermentation with selected LAB strains could lead to the better detoxication of some mycotoxins in vivo [34]. Finally, biocontrol of mycotoxins by LAB could be an important factor to reduce their risks during food fermentation.

The aim of this study was to analyze and compare the acidity, microbiological, and chromaticity parameters; fatty acid (FA) and volatile compound (VC) profiles; and biogenic amine (BA), macro- and microelement, and mycotoxin concentrations in nontreated and fermented (with antimicrobial/antifungal properties possessing LAB strains, isolated from spontaneous sourdough: *Pediococcus acidilactici*-LUHS29, *Liquorilactobacillus uvarum*-LUHS245, *Lactiplantibacillus plantarum*-LUHS122) ‘Ada’, ‘Sarta’, and new breed blue (DS8472-5) and purple (DS8526-2) wheat lines WW. Also, this study will contribute to the creation of the database of the grain chemical composition of the new breed wheat lines.

## 2. Materials and Methods

### 2.1. Wheat and Lactic Acid Bacteria Strains Used in the Experiments and Wheat Wholemeal Preparation and Fermentation

**Figure 1:**
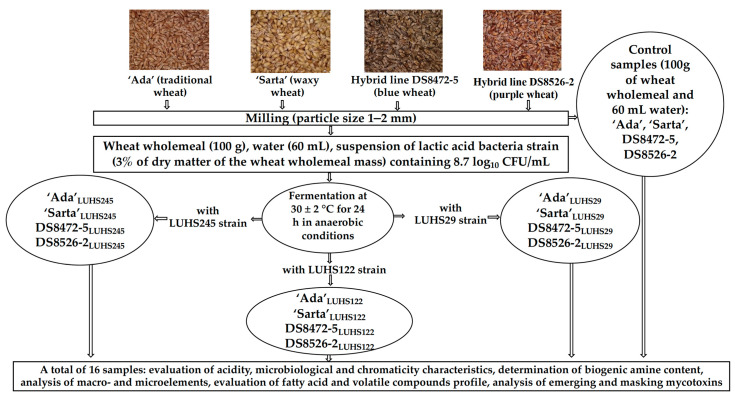
The principal scheme of the experiment (*Pediococcus acidilactici* LUHS29, *Liquorilactobacillus uvarum*—LUHS245, and *Lactiplantibacillus* plantarum—LUHS122).

The principal scheme of the experiment is given in Figure 1. The grains of wheat varieties ‘Ada’ (traditional wheat), ‘Sarta’ (waxy wheat), and new breed lines DS8472-5 (blue wheat) and DS8526-2 (purple wheat) were provided by the Institute of Agriculture, Lithuanian Research Centre for Agriculture and Forestry (LAMMC, Akademija, Kėdainiai distr., Lithuania). Field trials were conducted during 2020–2021 at the experimental base of the LAMMC in Akademija, Kėdainiai distr., Lithuania (55°39′ N 23°57′ E). Field experiments were designed in four replications (plot size 5 × 1.6 m), and each replication was grown in a separate block, where the field plots were randomly arranged. The trials were conducted under a sustainable growing technology. The soil was light loam Endocalcari-Epihypogleyic Cambisol. Topsoil (0–30 cm) pH was low acid (5.5), close to low in humus (1.6%), high in available phosphorus (251 mg/kg P_2_O_5_), and moderate in available potassium (175 mg/kg K_2_O). Winter wheat was sown with treated seeds at the seed rate of 4.5 million ha^−1^ in the beginning of the 10th of September (2020) after the black fallow. Complex mineral fertilisers (N_15_P_50_K_100_) were applied in the entire experimental field before sowing. Nitrogen fertilisers (ammonium nitrate) were applied after resumption of spring vegetation (on the 8 April 2021) and when plants reached the stem elongation stage (on the 4 May 2021). The rate of N_100+30_ was used. Weeds were controlled by the recommended herbicides in the autumn and spring. Yield was harvested on the 21 July 2021. The elevation of the experimental area is 82 m above sea level, belongs to the mid-latitude climate zone in the southwestern subregion of the Atlantic continental forest area. According to the data of the local Dotnuva Meteorological Station (55°23′49.0″ N 23°51′55.0″ E), the climatic conditions were characterized by the long-term (1924–2021) average annual temperature of 6.5 °C and precipitation of 570 mm. Both experiment growing seasons, especially the spring–summer period, were warmer (2.3 °C ). In addition, the 2020–2021 growing season was dryer than the long-term average. The autumn of 2020 was warmer and dryer than usual. The winters were quite hard, and thick snow cover protected the plants from cold damage but provoked intense development of snow mold. The spring meteorological conditions were variable—March was warmer and dryer, April was colder and dryer, and May was extremely cold and wet as precipitation was twice as much. The drought period was recorded from the middle of June to the end of July. June and July were warmer by 3.1 °C and 4.7 °C, respectively. The first part of July was warmer by 6.5 °C; this affected the rapid maturation of plants, which formed small grains. A wheat wholemeal (WW) was prepared by milling wheat grains (moisture content of the wheat was 14%) with Laboratory Mill 120 (Perten Instruments AB, Stockholm, Sweden) until the particles size was 1–2 mm. The moisture content of the grain was determined by using Infratec™ 1241 Grain Analyser (FOSS, Foss Allé DK-3400, Hilleroed, Denmark).

The LAB strains *Pediococcus acidilactici* LUHS29, *Liquorilactobacillus uvarum*-LUHS245, and *Lactiplantibacillus plantarum*-LUHS122, were used for WW fermentation. The LAB strains were obtained from the Lithuanian University of Health Sciences (Kaunas, Lithuania). Our previous studies showed, that the LUHS122 strain ferment 28, LUHS245—23, and LUHS183—20 out of 47 tested carbohydrates; LUHS122 and LUHS183 showed tolerance to 30, 37, and 45 °C and LUHS122 to 30 and 37 °C; the concentration of viable cells after 2 h incubation at pH 2.5 for LUHS122 was 5.72 ± 0.2 log_10_ CFU/mL, for LUHS245 was 7.55 ± 0.2 log_10_ CFU/mL, for LUHS183 was 3.20 ± 0.2 log_10_ CFU/mL [21].

Before the experiments, the LAB strains were stored at −80 °C (Microbank system, Pro-Lab Diagnostics, Birkenhead, UK) and multiplied in de Man–Rogosa–Sharpe (MRS) broth (Oxoid Ltd., Hampshire, UK) at 30 ± 2 °C for 24 h, before using them in the WW fermentation. The WW, water, and a suspension of LAB strain (3% of dry matter of the WW mass) containing 8.7 log_10_ CFU/mL were fermented at 30 ± 2 °C for 24 h in a chamber incubator (Memmert GmbH + Co. KG, Schwabach, Germany). For 100 g of WW, 60 mL of water was used. Nonfermented WW samples were analyzed as a control.

### 2.2. Evaluation of Acidity, Microbiological, and Chromaticity Characteristics of Wheat Wholemeal Samples

The pH of WW was measured using a pH electrode (PP-15; Sartorius, Goettingen, Germany). The total titratable acidity (TTA) was evaluated for a 10 g WW sample mixed with 90 mL of water; results were expressed as mL of 0.1 mol/L NaOH solution required to achieve a pH value of 8.2. The LAB count was determined according to the method described by Bartkiene et al. [35]. The chromaticity characteristics of WW were evaluated on the WW surface using a CIE L*a*b* system (CromaMeter CR-400, Konica Minolta, Japan). The results were expressed as the CIE color values L* (brightness/darkness), a* (redness/greenness), and b* (yellowness/blueness).

### 2.3. Determination of the Biogenic Amine Content in Wheat Wholemeal Samples Nonfermented and Fermented with Different Lactic Acid Bacteria Strains

The extraction and determination of BA in nonfermented and fermented WW followed the procedures developed by Ben-Gigirey et al. (1999) [36]. Perchloric acid (0.4 mol/L, 10 mL) containing a known amount of 1,7-diaminoheptane as an internal standard was added to 3 g of sample, and the mixture was homogenized with Ultra-Turrax apparatus (IKA Labortechnik, Staufen, Germany) and centrifuged at 3000 *g* for 10 min. The residue was extracted again with an equal volume of 0.4 mol/L perchloric acid. Both supernatants were combined, and the final volume was adjusted to 30 mL with 0.4 mol/L perchloric acid. The extract was filtered through Whatman No. 1 paper filter. One mL of extract or standard solution was mixed with 200 mL of 2 mol/L NaOH solution and 300 mL of saturated NaHCO_3_ solution. A 10 mg/mL solution of 5-(dimethylamino)naphthalene-1-sulphonyl chloride (dansyl chloride) (2 mL) in acetone was added to the mixture and incubated at 40 °C for 45 min. Residual dansyl chloride was removed by the addition of 25 mg/L aqueous ammonia solution (100 mL). After incubation at room temperature for 30 min, the mixture was adjusted to 5 mL with acetonitrile. Finally, the mixture was centrifuged at 3000× *g* for 5 min, the supernatant was then filtered through a 0.2 μm filter (Millipore, Bedford, MA, USA) and stored at 25 °C until HPLC analysis.

An Agilent 1200 HPLC system (Carlsbad, CA, USA) equipped with a diode array detector and Chemstation LC software was employed in combination with a Chromolith C_18_ HPLC column (100 mm × 4.6 mm × 4 μm, Merck KGaA/EMD Chemicals, Darmstadt, Germany). Ammonium acetate (0.1 mol/L) and acetonitrile were used as the mobile phases at the flow rate of 0.45 mL/min. The injected sample volume was 10 μL and the amines were monitored at 254 nm wavelength. The detection limits for standard amine solutions were approximately 0.1 mg/kg.

### 2.4. Analysis of Macro- and Microelements of Wheat Wholemeal Samples Nonfermented and Fermented with Different Lactic Acid Bacteria Strains Using Inductively Coupled Plasma Mass Spectrometry (ICP-MS)

For macro- and microelement analysis in WW samples, an Agilent 7700x ICP-MS (Agilent Technologies, Tokyo, Japan) and software MassHunter Work Station for ICP-MS, version B.01.01 (Agilent Technologies, Tokyo, Japan) were used. The samples were milled and homogenized (final particle size ≤ 150 µm). For the analysis the following chemicals were used: nitric acid (concentration ≥ 69.0%), for trace analysis (Saint Quentin-Fallavier, Sigma–Aldrich, Saint Quentin-Fallavier, France), hydrogen peroxide, 30% *w*/*w* (weight/weight), extra pure (Scharlau, Barcelona, Spain), multielement standard solution V for ICP-MS calibration (Sigma–Aldrich, France). Agilent 7700x ICP-MS (Agilent Technologies, Tokyo, Japan), software MassHunter Work Station for ICP-MS, version B.01.01 (Agilent Technologies, Tokyo, Japan) were used for analysis. Sample preparation for ICP-MS analysis was carried out as follows: 0.3 g of the WW was accurately weighed in a microwave vessel; then, 2 mL of de-ionized water, 8 mL of concentrated nitric acid, and 2 mL of concentrated hydrogen peroxide were added. This was left for 2–8 h for reaction stabilization until the formation of bubbles was finished. The vessel was sealed and heated in the microwave system. The following thermal conditions were applied: 150 °C was reached in approximately 20 min and remained for 30 min, and then 200 °C was reached in approximately 20 min and remained for 30 min for the completion of specific reactions. After cooling (approximately 40 min), the prepared solution was filtered through the filter with a pore size 8–10 μm. The solution was transferred to the 50 mL volumetric flask and filled with water to 50 mL volume. The following operating conditions of Agilent 7700x ICP-MS were used for the analysis of the samples: plasma mode—normal, robust; RF forward power 1300 W; sampling depth 8.00 mm; carrier gas flow 0.6 L/min; dilution gas flow 0.4 L/min; spray chamber temperature 2 °C; extraction lens 1 V; kinetic energy discrimination 3 V.

### 2.5. Fatty Acid Composition Analysis

The fatty acid (FA) composition of the WW samples was determined using GCMS-QP2010 (Shimadzu, Kyoto, Japan) gas chromatograph with a mass spectrometer. The fatty acid methyl esters (FAME) concentration was determined using a calibration curve and results were expressed as the percentage of total FAME concentration in the sample. The sample was prepared by homogenizing 1 g of WW sample in 5 mL of 30% (*w*/*v*) NaCl solution. Next, 5 mL of hexane were added. The mixture was shaken on a laboratory shaker for 1 h. The mixture was centrifuged at 4000 rpm. Afterwards 4 mL of the hexane extract were reacted with 300 µL of methylation reagent (2 mol/L of KOH in methanol) by vortexing and shaking using a laboratory shaker for 1 h. The mixture was centrifuged at 4000 rpm and the upper layer was filtered using a 0.22 µm membrane syringe filter and used for the analysis. Capillary Stabilwax-MS column (30 m × 0.25 mm ID × 0.25 µm film thickness. Mass spectrometer operated at full scan mode. Analyte was injected in split mode at 1:60 split ratio. The following parameters were used: MS ion source temperature: 240 °C, MS interface temperature 240 °C, helium (carrier gas) flow: 0.90 mL/min, injector: 240 °C, oven temperature 50 °C (4 min), 10 °C/min to 110 °C (1 min), 15 °C/min to 160 °C (2 min), 25 °C/min to 195 °C (1 min), 2 °C/min to 230 °C (1 min), 2 °C/min to 240 °C (12 min).

### 2.6. Evaluation of Volatile Compound Profile

The VCs of the WW samples were analyzed by gas chromatography-mass spectrometry (GC-MS). A solid phase microextraction (SPME) device with a Stableflex™ fibre coated with a 50 µm PDMS-DVB-Carboxen™ layer (Supelco, St. Louis, USA) was used for the analysis. A WW sample was weighed and blended with an aqueous NaCl solution (30% *w*/*v*) in a ratio of 1 g of WW to 3 mL of NaCl solution. For headspace extraction, 8 g of prepared sample was transferred to the 20 mL extraction vial, sealed with a polytetrafluoroethylene septum, and thermostatted at 60 °C for 15 min before exposing the fibre in the headspace. The fibre was exposed to the headspace of the vial for 10 min and desorbed in an injector liner for 2 min (splitless injection mode). Prepared samples were analyzed with a GCMS-QP2010 (Shimadzu, Kyoto, Japan) gas chromatograph and mass spectrometer. The following conditions were used for the analysis: injector temperature of 250 °C, ion source temperature of 220 °C, interface temperature of 260 °C. Helium was used as the carrier gas at a flowrate of 0.95 mL/min. A Stabilwax-DA capillary column (0.25 mm ID, 0.25 μm film thickness, 30 m length (Restek, Bellefonte, PA, USA)) was used for the analysis. The temperature gradient was programmed from a starting temperature of 40 °C (3 min hold) to 220 °C (6 °C/min) to 250 °C (10 °C/min) (6 min hold). The VCs were identified according to mass spectrum libraries (NIST11, NIST11S, FFNSC2).

### 2.7. High-Performance Liquid Chromatography Coupled to Triple Quadrupole Mass Spectrometry (HPLC-MS/MS) for Mycotoxin Analysis

#### 2.7.1. Materials and Chemicals

Mycotoxin standards were of at least 95% purity. Standards were supplied by Romer Labs (Tulln, Austria) (Ochratoxin A, Fumonisin B1, Fumonisin B2, T2-Toxin, HT-2 Toxin, Zearalenone, Deoxynivalenol) and Fermentek (Jerusalem, Israel) (Aflatoxin B1). HPLC grade acetonitrile and methanol (>99% assay), ACS grade formic acid (≥96.0% assay) was used. Ultrapure water (18.2 MΩ × cm) was generated by a Milli-Q system (Millipore, Billerica, MA, USA). QuEChERS buffer–salt extraction kits consisting of magnesium sulphate (4 g), sodium chloride (1 g), trisodium citrate dihydrate (1 g), and disodium hydrogen citrate sesquihydrate (0.5 g) per portion and QuEChERS dSPE kit consisting of magnesium sulphate (900 mg), PSA (primary and secondary amines) (150 mg) and C18E (150 mg) per 15 mL polypropylene tubes were purchased from Phenomenex (Torrance, CA, USA). Unprocessed wheat was used as a blank sample.

#### 2.7.2. Sample Preparation

Samples (2.50 ± 0.01 g) were accurately weighed in 50 mL PP tubes. The quality control (blank) samples were spiked with mycotoxin standard solutions at the appropriate spiking levels. Then water (10 mL), acetonitrile (10 mL), and formic acid (20 µL) were gradually added to the tubes and extraction was started by mixing for 10 min on mechanical shaker. One portion of the QuEChERS buffer salt kit was added to each of the tubes and the extraction was continued for additional 10 min. The obtained mixtures were centrifuged (4500 rpm, 10 min). The supernatants were transferred to 15 mL centrifuge tubes and placed for 15 min at −80 °C in a Heto PowerDry^®^ freeze dryer (Thermo Fisher Scientific, Waltham, MA, USA). After removal, the extracts were immediately centrifuged (4000 rpm, 15 min) at 15 °C, and 500 µL of the upper layer were transferred to 15 mL centrifuge tubes. Then, 6 mL of the remaining extract were transferred to QuEChERS dspe kit, then mixed for 5 min on a mechanical shaker and centrifuged (3000 rpm, 10 min). Next, 3500 µL of supernatant were combined with 500 µL from before and then the combined extracts were evaporated to dryness at 50 °C under a gentle nitrogen stream. The dry residues were reconstructed in 300 µL 0.1% formic acid in water/methanol (6/4*_v_*_/*v*_). Extracts were filtered through 0.22 µm Millipore UltraFree MC centrifuge filters (3900× *g*, 10 min) and transferred into the autosampler for analysis.

#### 2.7.3. Chromatographic Method

The analysis was performed on an UltiMate™ 3000 (Thermo Fisher Scientific, Waltham, MA, USA) HPLC coupled with a Thermo Scientific TSQ Quantiva MS/MS detector. The separation was performed on a Kinetex 1.7 µm C18 reversed-phase analytical column (50 × 3.0 mm, 1.7 µm). The autosampler was maintained at 15 °C and the column temperature was 40 °C. The sample injection volume was 15 µL. Ion monitoring was conducted in both positive and negative ion modes and the mass analysis was performed in selected reaction monitoring (SRM) mode. The following instrumental settings were used: spray voltage 3.5 kV (positive ion mode), 2.5 kV (negative ion mode), vaporiser temperature 350 °C, ion transfer temperature 300 °C, sheath gas 55 arbitrary units (arb), auxiliary gas 25 arb, and sweep gas 5 arb. Data processing was performed with TraceFinder software (Thermo Fisher Scientific). More detailed information on parameters of HPLC and MS/MS is provided in Supplementary File S1 Tables S1 and S2.

Six-point calibration curves were constructed using blanks spiked with mycotoxin standard mixtures (1 μg/kg (or LOQ) to 250 μg/kg). The least squares regression method was used for slope construction and calculation of the determination coefficients (R2) of the calibration curves, which were evaluated to a fit of at least 0.99.

### 2.8. Statistical Analysis

Microbiological results were expressed as the mean (*n* = 5) ± standard error (SE), and physico-chemical results were expressed as the mean (*n* = 3) ± standard error (SE). The experiment was performed by preparing three parallel samples for fermentation. In order to evaluate the effects of the different wheat varieties and different LAB strains used for WW fermentation, the data were analyzed by multivariate analysis of variance (ANOVA). To determine the normality the Shapiro–Wilk Test was used, for homoskedasticity evaluation the homoskedasticity test by using SPSS Statistical Package was performed. The linear Pearson’s correlation coefficients were calculated using the statistical package SPSS for Windows (v15.0, SPSS, Chicago, IL, USA). Correlation strength interpretation was performed in accordance with the procedure by Evans (1996) [37]. The results were recognized as statistically significant at *p* ≤ 0.05.

## 3. Results and Discussion

### 3.1. Acidity, Chromaticity, and Microbiological Characteristics of the Wheat Wholemeal Samples

The acidity, chromaticity, and microbiological characteristics of the WW samples are shown in Table 1. When comparing the acidity parameters (pH and TTA) of the fermented samples, it was established that the type of LAB used for fermentation was a significant factor in sample pH and TTA (*p* = 0.0001 and *p* = 0.011, respectively) (Supplementary File S2 Table S3). However, the wheat variety and the wheat variety * LAB type interaction was not a significant factor in the acidity parameters of the samples. Between the pH and TTA, a negative moderate correlation was found (r = −0.422, *p* = 0.003) (Supplementary File S2 Table S4). When comparing the pH of the fermented samples in different wheat variety groups, in ‘Ada’ and DS8472-5 blue WW, the lowest pH was found after fermentation with the LUHS122 strain (on average, pH 3.97). Also, in the ‘Sarta’ WW group, the lowest pH was found in the samples fermented with LUHS245 and LUHS122 strains (on average, pH 3.83). Opposite tendencies of the DS8526-2 purple WW were established, and the lowest pH of the DS8526-2 fermented with LUHS29 was obtained (pH 4.24). When comparing the chromaticity characteristics of the samples, the wheat variety * LAB type interaction was a significant factor in the sample redness (a*) (*p* = 0.004) (Supplementary File S2 Table S3); however, significant correlations between the a* and acidity parameters were not found (Supplementary File S2 Table S4). WW lightness (L*) and yellowness (b*) showed moderate positive correlations with sample TTA (r = 0.578, *p* = 0.0001 and r = 0.341, *p* = 0.018, respectively) (Supplementary File S2 Table S4), and when comparing the sample L* in non-treated and fermented different variety WW groups, different tendencies were established. In the ‘Ada’ and DS8526-2 purple WW group, fermented samples showed a decrease in the L* coordinates (in comparison with the nonfermented ones, on average, by 8.7% and 14.1%, respectively). Opposite tendencies in the ‘Sarta’ sample group were found, in which L* coordinates increased after fermentation (in comparison with the nonfermented ones, on average, by 12.6%). The L* of the DS8472-5 blue WW samples was dependent on the LAB strain used for fermentation: the LUHS29 strain decreased the L* (on average, by 14.1%), and fermentation with LUHS245 and LUHS122 increased the L* (on average, by 5.6%). When comparing the a* and b* coordinates of the nontreated and fermented samples, fermentation with all three tested LAB strains reduced the a* and b* parameters of the DS8526-2 purple WW, and the same tendencies were found for the a* coordinates of the fermented ‘Sarta’ samples. However, fermentation increased the a* coordinates of the DS8472-5 blue WW and b* coordinates of the ‘Sarta’ samples. The chromaticity parameters of the other fermented samples were varied and depended on the LAB strain used for fermentation and the wheat variety.

Anthocyanin, flavonoids, carotenoids, and some other phenolics are responsible for the different colors in wheat [38]. Anthocyanins are located in the cereal pericarp and account for the blue, purple, or a combination of both colors depending on its concentration [5]. It was reported that the anthocyanin concentration could be reduced during the fermentation process [39]. The yellow color of cereal is related to the presence of carotenoids, and the red color is associated with the presence of phlobaphenes [40]. It was reported that an increase in the carotenoid content can be obtained in fermented doughs, and these changes were explained by an increased mobilization of membrane-associated lipophilic compounds from the cereal matrix; however, this process is strain-specific [41]. In addition, the changes in the lutein and zeaxanthin content in doughs fermented by the different LAB strains are variable and could be due to the higher degradation of this compound during fermentation, probably because of the higher lipid oxidation involving the endogenous lipoxygenase/linoleate system [42]. Also, LAB could possess strong antioxidative effects, through a decrease in the oxidation-reduction potential of sourdough [43], and, conversely, to reduce oxidation of the color compounds.

It was reported that all of the LAB strains belonging to the genera Lactobacillus as well as P. pentosaceus acidified the medium at around pH 3.5 [44]. When comparing the LAB count in the fermented samples, all the fermented WW showed higher than 8.0 log_10_ CFU/g of the LAB number, and a moderate negative correlation between the LAB count and sample pH was established (r = −0.406, *p* = 0.004) (Supplementary File S2 Table S4). The main factors that lead to the competitiveness of technological LABs in fermented cereal are fast LAB multiplication in a substrate as well as increases in TTA and decreases in pH [45]. According to other studies, a lower LAB number and acidification rate are obtained in homofermentative LAB fermented cereal in comparison with heterofermentative ones [46]. However, the acidification rate is related to many factors (variety of cereal, type of flour, fermentation temperature, acidification of LAB strain, moisture content, duration of the fermentation process, etc.) [47]. The mechanisms in cereal fermentation are very complex [48]. During fermentation, in addition to biochemical changes that occur in the carbohydrate of the flour, changes in the protein components also take place due to the action of both microorganisms and indigenous flour enzymes [49], as well as endogenous factors in the cereal substrate (free fatty acids, minerals, carbohydrates, nitrogen sources, enzyme activities, etc.) [48]. Also, it should be taken into consideration that the bran fraction contains more minerals and other micronutrients that improve the effectiveness of LAB growth [47]. In addition, the ash has an influence on the buffering capacity of the fermentable substrate; for this reason, it is possible to reach a higher TTA. This study showed that the LAB type is a significant factor in the LAB count in the samples (*p* = 0.0001); however, the wheat variety and wheat variety * LAB type interaction were not a significant factor in the LAB number in the samples (Supplementary File S2 Table S3).

### 3.2. Biogenic Amine Content in Wheat Wholemeal Samples

The biogenic amine (BA) content in WW samples is shown in Table 2. Tryptamine (TRY) and spermine (SPRM) were not found in WW; however, all the other BAs identified in the WW samples were significantly influenced by the wheat variety, the type of LAB used for fermentation, and the wheat variety * LAB type interaction (Supplementary File S3 Table S5). Also, between the sample pH and phenylethylamine (PHE), a positive weak correlation was found (r = 0.389, *p* = 0.006). Also, between the LAB count in the samples and the spermidine (SPRMD) concentration, a positive moderate correlation was established (r = 0.422, *p* = 0.003) (Supplementary File S3 Table S6). PHE was the predominant BA in the WW samples, and the lowest PHE concentration was found in the DS8526-2 purple WW samples fermented with the LUHS245 strain (26.4 mg/kg). In the other samples, the PHE concentration was from 2.8 to 1.8 times higher (in nontreated ‘Ada’ WW and in LUHS245 fermented ‘Sarta’ samples, respectively). Putrescine (PUT) was established in all (nontreated and fermented) ‘Ada’ and ‘Sarta’ samples. When comparing the PUT concentration in the ‘Ada’ samples, the lowest PUT content was found in the LUHS29 fermented ‘Ada’ WW (20.3 mg/kg). However, in nontreated and in LUHS245 fermented ‘Ada’ samples, on average, a 40.9% higher PUT concentration was established, and the highest PUT content was formed in the LUHS122 fermented samples (98.1 mg/kg). PUT was not found in DS8472-5 blue WW, and when comparing DS8526-2 purple WW, PUT was formed in just one sample (fermented with the LUHS245 strain) (26.8 mg/kg). Cadaverine (CAD) was not found in waxy wheat ‘Sarta’ WW and in other wheat varieties WW fermented with the LUHS29 strain. Also, CAD was not established in DS8472-5 blue WW fermented with the LUHS245 strain; however, in DS8526-2 purple WW fermented with the LUHS245 strain, the CAD concentration was, on average, 84.0 mg/kg. In both blue and purple WW fermented with the LUHS122 strain, on average, a CAD concentration of 94.0 mg/kg was found. Histamine (HIS) was found in just two samples (in ‘Ada’ and ‘Sarta’ WW fermented with the LUHS122 strain), and tyramine (TYR) was found in one sample (DS8526-2 purple WW fermented with the LUHS122 strain). The spermidine (SPRMD) concentration in WW samples ranged from, on average, 28.0 mg/kg (in nonfermented and LUHS245 fermented ‘Ada’, in LUHS245 fermented ‘Sarta’, in LUHS29 and LUHS122 fermented blue WW, and in all purple WW samples) to, on average, 36.3 mg/kg (in LUHS29 fermented ‘Ada’ WW and in LUHS245 fermented blue WW).

HIS, TYR, PUT, CAD, TYR, agmatine, SPRM, and SPRMD are the main BAs in food and beverages [50,51]. Despite the fact that the LAB has a GRAS (Generally Recognized as Safe) status, these very popular industrial microorganisms are the main BA producers in fermented foods [52]. In addition, it was reported that the pH between 4.0 and 5.5 is optimal for enzymes, forming BAs, activities [50]. Other factors that influence the formation of BAs are bacterial activity, humidity, the concentration of free amino acids, etc.; however, the number of bacterial cells in the substrate does not always correlate with the amount of BAs [53]. Consumption of food containing a high concentration of BAs is implicated in various pharmacological reactions [54], which lead to different types of foodborne diseases [27]. HIS can be formed from histidine, TYR from tyrosine, CAD from lysine, PUT from arginine, and SPRMD and SPRM from arginine and methionine, and their toxicity depends on synergistic effects, e.g., HIS toxicity is enhanced by the presence of CAD, PUT, and TYR [55]. Despite the fact that HIS and TYR are the most toxic BAs [56,57,58,59], PUT and CAD, although not toxic themselves, aggravate the adverse effects of HIS and TYR [60]. Until now, the European legislation does not specify a BA threshold; however, the European Food Safety Authority (EFSA) reported a scientific opinion on the risk associated with the formation of BAs in fermented products [29]. Based on the mean content in foods and consumer exposure data, fermented food categories were ranked with respect to HIS and TYR, but other BAs were not included. Finally, further research on BAs in fermented foods is needed, and, as this study indicated, there is a significant influence of the wheat variety, the type of LAB used for fermentation, and the wheat variety * LAB type interaction on the formation of BAs in fermented WW.

### 3.3. Macro- and Microelements of Wheat Wholemeal

Macroelement concentrations in the WW samples are shown in Table 3. When comparing the Na concentration in the WW samples, the Na concentration was higher in all the fermented WW compared to the nontreated WW. Taking into consideration that LAB multiplication was performed in a medium (MRS broth) containing sodium acetate, it could be that the LAB strains were the source of Na in the WW samples. The wheat variety and LAB strain used for fermentation were not significant factors on the Mg and K concentrations in WW; however, for the Ca concentration, the wheat variety was a significant factor (*p* = 0.002).

Essential microelement concentrations in the WW samples are shown in Table 4. In all the tested samples, the Co concentration was <0.010 mg/kg, the Ni concentration was <0.500 mg/kg, and the Se concentration was <0.200 mg/kg. Also, the Cr concentration was <0.010 mg/100 g in most of the samples except the ‘Ada’ WW fermented with LUHS122, nontreated and fermented with LUHS29 ‘Sarta’, and nontreated DS8472-5 blue wheat WW. Both the wheat variety and LAB strain used for fermentation were significant factors on the Mn concentration in the WW samples (*p* = 0.001). When comparing the nonfermented samples, the highest Mn content was found in the DS8526-2 purple WW (15.3 mg/kg), and other varieties showed similar Mn concentrations (on average, 9.77 mg/kg). Also, the wheat variety and LAB strain used for fermentation were significant factors on the Fe concentration in the WW samples (*p* = 0.003). When comparing the nontreated samples, ‘Ada’ WW showed the highest Fe concentration (on average, 34.1% higher, in comparison with ‘Sarta’, DS8472-5, and DS8526-2 varieties), and Zn concentration in nontreated WW was, on average, 7.27 mg/kg. Wheat variety was a significant factor in the Cu concentration in WW (*p* = 0.001). When comparing nontreated WW samples, the highest Cu concentration was found in ‘Ada’ WW (1.85 mg/kg) and the lowest in DS8472-5 blue WW (0.763 mg/kg).

Non-essential microelement concentrations in the WW samples are given in Table 5. The wheat variety and LAB strain used for fermentation were significant factors on the Sr concentration in the WW samples (*p* = 0.018 and *p* = 0.041, respectively). When comparing nontreated WW, a higher concentration of Sr in both colored wheat was found in comparison with traditional and waxy wheat (on average, by 33.9%). Also, the wheat variety was a significant factor in the Cd concentration in WW (*p* = 0.41). Pb was found only in ‘Ada’ WW. Most of the other non-essential microelements in the WW samples were below the detection limits.

Wheat grain could be a very important source of micro- and macroelements in the human diet [61]; for this reason, it is very important to have a database of the detailed chemical composition of wheat grains. Also, the concentration of trace elements is a very important factor in wheat-breeding programs focused on obtaining plants resistant to environmental stress [62]. However, the mineral content and their bioavailability can change during the fermentation of cereal [63]. Minerals have a significant role in microorganism growth and their enzyme-excreting properties, as well as the enzyme activity [64]. It was reported that Mn^2+^, Mg^2+^, Ca^2+^, Fe^2+^, K^+^, and Na^+^ are essential for the nutrient metabolism and enzymatic activity of LABs [65]. From this point of view, they can affect the main processes of the bread preparation, especially sourdough bread production. The colored wheat breed has been reported as a specific micronutrient source, of which the organic Cr content is four times higher than that of common wheat [66]. Also, according to He and Ning, black wheat “Qinhei 1” showed the Fe and Zn contents by 19.2 and 4.1 times higher, respectively, than that of common wheat, and the contents of Mn, Cu, Se, Mg, K, and P also were higher than that of common wheat [67]. It was reported that the Zn, Fe, and Mg content in colored wheat is, on average, from 108.54 to 142.68%, from 8.57 to 42.86%, and from 5.31 to 40.63% higher than that in common wheat, respectively [68]. These results may be related to the lower yield of colored wheat. According to Tian, Chen, and Wei, blue wheat has the highest Ca content [68]. However, published studies on the micro- and macroelements in various wheat varieties are varied. It was reported that the fertilizers that are used are a significant factor in the concentration of these compounds, i.e., fertilizers increases the Fe content in the red wheat variety (68.88 mg/kg) and the Zn content in the common variety (40.43 mg/kg); however, the same fertilizer was not so effective on the Fe and Zn content in the purple wheat variety (Fe—55.59 mg/kg, Zn—38.50 mg/kg) [69]. Finally, the development of the database of new wheat cereal breed lines is very important because of the efficient use of the cereal in food and/or feed industries.

### 3.4. Fatty Acid Composition of the Wheat Wholemeal Samples

**Figure 2:**
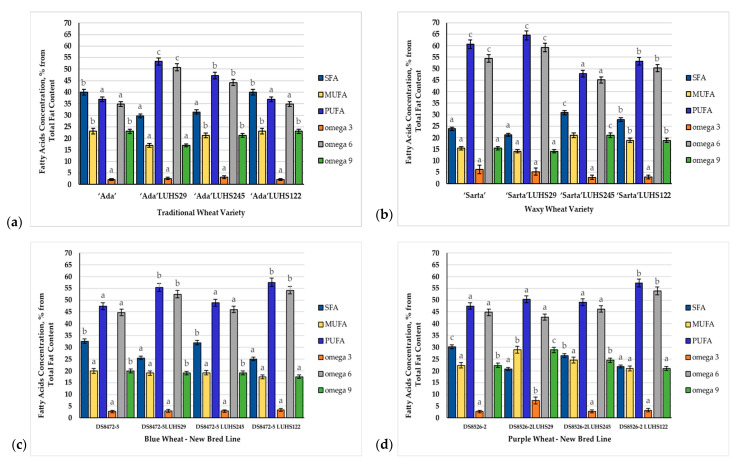
Fatty acid profiles of wheat wholemeal samples: (**a**) Traditional wheat variety fatty acid profile (**b**) Waxy wheat variety fatty acid profile (**c**) Blue wheat—new breed line fatty acid profile (**d**) Purple wheat—new breed line. LUHS29—fermented with *Pediococcus acidilactici* LUHS29 strain; LUHS245—fermented with *Liquorilactobacillus uvarum* LUHS245 strain; LUHS122—fermented with *Lactiplantibacillus plantarum* LUHS122 strain; SFA—saturated fatty acids, MUFA—monounsaturated fatty acids, PUFA—polyunsaturated fatty acids. Data are represented as means (*n* = 3) ± SE. ^a–c^ Means with different letters in the column are significantly different between the same variety of non-treated and fermented samples (*p* ≤ 0.05).

The saturated (SFA), monounsaturated (MUFA), polyunsaturated (PUFA) fatty acids, omega 3, omega 6, and omega 9 composition of the WW samples is shown in Figure 2, and the detailed FA profile is given in Supplementary File S4 Table S7. The main FAs in WW were palmitic acid (C16:0), all-*cis,trans*-octadecenoic acid (C18:1), and linoleic acid (C18:2). The type of LAB used for fermentation was a significant factor in the C18:1 content in WW (*p* = 0.033). Also, the wheat variety, LAB type used for fermentation, and their interaction were significant factors on most of the FA content except C18:3 α (alfa linolenic acid) (Supplementary File S4 Table S8). When comparing ‘Ada’ WW, in all the cases, in nontreated WW and WW fermented with the LUHS122 strain, SFA, MUFA, PUFA, omega 3, omega 6, and omega 9 quantities were similar (on average, 39.95, 23.12, 36.93, 2.11, 34.82, and 23.12%, respectively). However, in WW samples fermented with LUHS29 and LUHS245 strains, SFA, MUFA, and omega 9 were decreased (in comparison with nontreated ones, on average, by 23.4, 17.4, and 17.34%, respectively), and PUFA, omega 3, and omega 6 were increased (in comparison with nontreated ones, on average, by 36.1, 36.0, and 36.3%, respectively). When comparing nontreated and fermented waxy wheat ‘Sarta’ samples, fermentation with LUHS29 decreases SFA, MUFA, and omega 3 and 9 and increases PUFA and omega 6 in WW. Opposite tendencies (except omega 3) were found in samples fermented with LUHS245 and LUHS122, in which an increase in SFA, MUFA, and omega 9 was found, as well as a decrease in PUFA, omega 3 and 6. When comparing nontreated and fermented DS8472-5 blue WW, all the tested LAB strains showed the same tendency of decreasing SFA, MUFA, and omega 9 and increasing PUFA, omega 3, and omega 6. Different tendencies of the FA in DS8526-2 purple WW were found. All the fermented samples showed lower SFA and higher PUFA and omega 3 content; however, fermentation with LUHS29 and LUHS245 increases MUFA and omega 9, and fermentation with LUHS122 showed opposite tendencies: decrease in MUFA and omega 9 in the WW samples. Also, fermentation with LUHS29 decreases omega 6 in the purple WW samples and increases omega 6 in comparison with the nonfermented ones.

It was reported that the wheat kernels grains contain, on average, 2.4–3.8% dry basis of lipids [70]. The lipid location by botanical wheat part is followed: on average, 66% of lipids are located in germ, 15% in the bran, and 20% in the endosperm [71]. According to Narducci et al., wheat lipids consist of mostly unsaturated FAs, and linoleic and linolenic FAs are essential [71]. The above mentioned FAs are precursors of prostaglandins and membrane phospholipid molecules in the human body and are involved in regulation processes of the blood lipid [70,72,73]. However, the lipid content in wheat cereal and their composition is related to many factors [70,74,75]. It was reported that cold weather increases the lipid content in wheat [76]. Also, biotic and abiotic stresses have an influence on the FA composition in wheat [77]. In addition, different analytical methods can also be a factor that influences the differences in the FA profile [70,78]. It was reported that in the FA profile of wheat cereal, more than 60 peaks were identified, and the major components were saturated and unsaturated C16 and C18 and particularly C16:0, C18:1, and C18:2; however, C16:0, C18:0, C18:1, C18:2, and C18:3 were mentioned as the most important FAs in durum wheat [70]. The differences in the FA composition in wheat cereal are explained by the differences in genetic characteristics, pedoclimatic conditions, agronomical treatments, and analytical procedures [71]. It was reported that the interaction of genotype × year × treatment is the main factor that contributes to the variability in the FA in durum wheat [75]. In addition, the differences in SFA and UFA within the same variety could be associated with various kinds of biotic and abiotic stresses [76,77]. Finally, the knowledge of this research will be very useful in updating common and new lines wheat cereal composition databases in general.

### 3.5. Volatile Compound Profile of the Different Wheat Wholemeal Varieties

**Figure 3:**
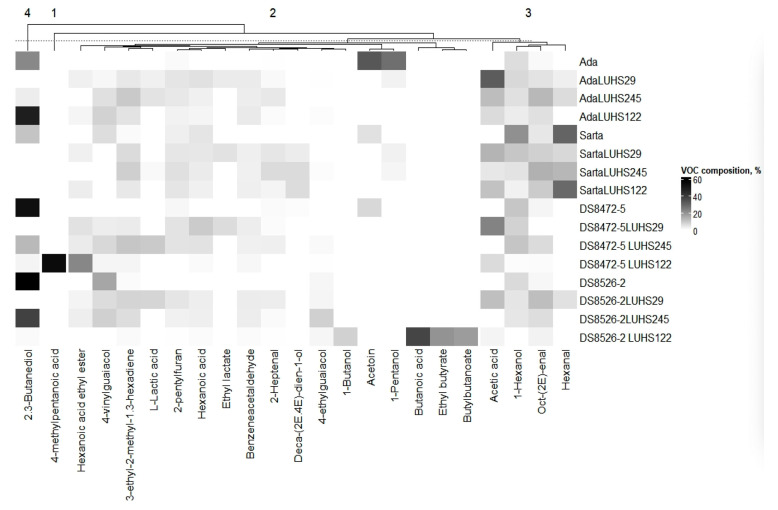
Volatile compounds that are >5% in at least one wheat wholemeal sample (LUHS29—fermented with *Pediococcus acidilactici* LUHS29 strain; LUHS245—fermented with *Liquorilactobacillus uvarum* LUHS245 strain; LUHS122—fermented with *Lactiplantibacillus plantarum* LUHS122 strain. Data are represented as means (*n* = 3)).

Volatile compounds (VCs) that were >5% in at least one WW sample are shown in Figure 3, and the detailed VC profile is given in Supplementary File S5 Table S9. When comparing acetic acid in the WW samples, it was not established in all the nonfermented samples, as well as in both color WW samples fermented with the LUHS245 strain. The odor of acetic acid is described as sharp, pungent, sour, and vinegary. However, in small concentrations, acetic acid is a flavor enhancer [15]. Heterofermentative LAB produces acetic acid, as well as specific esters, and reduces aldehydes to alcohols [79,80]. In purple WW fermented with the LUHS122 strain, 1-butanol was found (8.03% of the total VC). The odor of 1-butanol is described as fusel, oily, sweet, balsamic, and like whiskey. Acetoin, whose odor is described as creamy, dairy, sweet, oily, milky, buttery and like yogurt, was found only in nonfermented samples except purple WW. 1-Pentanol, which possesses a pungent, fermented, bready, yeasty, fusel, winey, and solvent-like odor, was one of the main compounds of nonfermented ‘Ada’ WW (27.7% of the total VC). Also, 2,3-butanediol (fruity, creamy, buttery odor) was found in all nonfermented WW, and its concentration changed in relation to the LAB strain used for fermentation. When comparing nonfermented samples, hexanal was found only in ‘Sarta’ WW (29.9% of the total VC); also, after fermentation, this VC was established in LUHS29 fermented ‘Ada’, ‘Sarta’, and purple WW (2.67, 6.66, and 5.51% of the total VC, respectively), in LUHS245 strain fermented ‘Ada’ and ‘Sarta’, and in LUHS122 strain fermented ‘Sarta’ WW. The hexanal concentration was 28.8% of the total VC. The odor of hexanal is described as aldehydic, fatty, green, and powerful. It was reported that the VC in the sourdoughs varied in relation with the starter culture used [81]. According to Hammes and Gänzle (1998) and Corona et al. (2016), the main VCs in wheat sourdough are acetic acid, hexanal, and octenal [48,82]. The lipid oxidation is the main factor for hexanal formation [18]. A high concentration of hexanal is undesirable, and the heterofermentative LAB could reduce its negative flavor by converting aldehydes to alcohols [18,83,84]. It was reported that hexanol is present in all fermentations [15]. Ethyl butyrate (sweet, fruity, tutti frutti, lifting, and diffusive odor) was found only in LUHS122 fermented purple WW (19.9% of the total VC). Ethyl lactate (sweet, fruity, acidic, etherial with a brown nuance odor) was found only in LUHS29 fermented ‘Ada’, ‘Sarta’, and blue WW (3.61, 5.05, and 5.99% of the total VC, respectively). Butanoic acid (sharp, dairy-like, cheesy, buttery with a fruity nuance odor) was one of the main VCs in LUHS122 fermented purple WW (37.7% of the total VC), while 1-Hexanol (citrus, fresh, floral, oily, sweet odor) was established in most of the samples except in purple WW fermented with the LUHS122 strain. L-lactic acid (sugar, wheat, malt, sweet odor) was found in ‘Ada’ and purple WW fermented with the LUHS29 strain and in ‘Ada’, ‘Sarta’, and blue WW fermented with the LUHS245 strain. More than half of the entire VC profile in blue WW fermented with the LUHS122 strain was 4-methylpentanoic acid (54.8% of the total VC), whose odor is described as pungent cheese. Furthermore, 2-Heptenal (intense green, fatty, oily, with fruity overtones odor) was found in most of the WW samples except in nonfermented ‘Sarta’, purple WW, and blue WW fermented with the LUHS122 strain. Hexanoix acid, whose odor is described as sour, fatty, sweat, and like cheese, was not formed in nonfermented ‘Ada’ and both colored WW samples, while 2-Pentylfuran (fruity, green, earthy beany with vegetable like nuances odor) was not established in only two WW samples (blue WW fermented with the LUHS122 strain and nontreated purple WW). Butylbutanoate (fruity, banana, pineapple, green cherry, tropical fruit, ripe fruit, and juicy fruit odor) was typical for purple WW fermented with the LUHS122 strain (17.9% of the total VC). The highest content of hexanoic acid ethyl ester (sweet, fruity, pineapple, waxy, green, banana odor) was found in LUHS122 fermented blue WW (22.4% of the total VC), and the LUHS245 fermented ‘Ada’ and blue WW had the highest content of 3-ethyl-2-methyl-1,3-hexadiene (on average, 9.50% of the total VC). Benzeneacetaldehyde (green, sweet, floral, honey, and cocoa odor) was typical for all the fermented samples, and oct-(2E)-enal (fresh, cucumber, fatty, green, herbal, banana, waxy, and green leaf odor) was found in most of the samples except in LUHS29 fermented blue WW. LUHS245 fermented purple WW had the highest content of 4-ethylguaiacol (spicy and clove-like with medicinal, woody, and sweet vanilla nuances odor) (8.27% of the total VC). Deca-(2E,4E)-dien-1-ol was abundant only in ‘Sarta’ WW fermented with all the tested LAB strains, and a very small amount of this compound was established in nonfermented blue WW. The odor of Deca-(2E,4E)-dien-1-ol is described as fatty and waxy, like white meat chicken and turkey with a slight melon fruity and a dairy nuance. The highest content of 4-vinylguaiacol (sweet, spicy, clove, carnation, phenolic, peppery, smoky, woody, powdery odor) was found in the VC profile of nonfermented purple WW (16.2% of the total VC).

**Figure 4:**
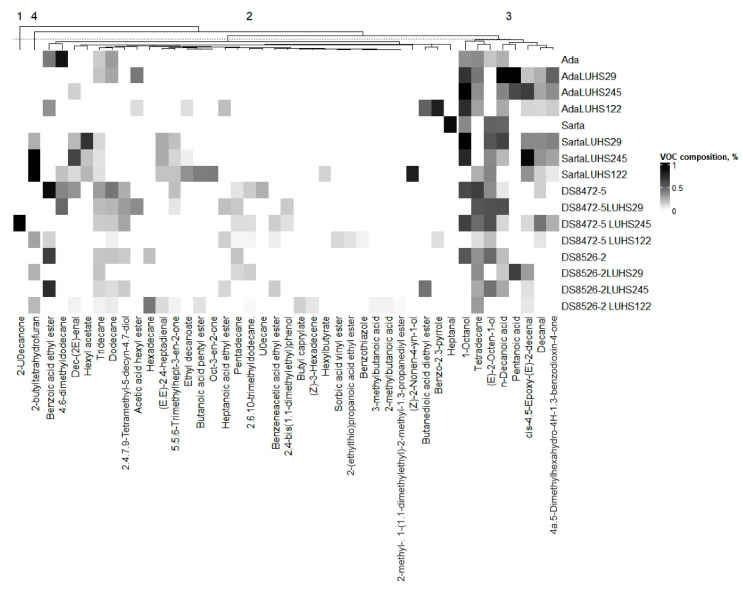
Volatile compounds that are >1% and <5% in at least one wheat wholemeal sample (LUHS29—fermented with *Pediococcus acidilactici* LUHS29 strain; LUHS245—fermented with *Liquorilactobacillus uvarum* LUHS245 strain; LUHS122—fermented with *Lactiplantibacillus plantarum* LUHS122 strain. Data are represented as means (*n* = 3)).

VCs that were >1% and <5% in at least one WW sample are shown in Figure 4, and the detailed FA profile is given in Supplementary File S5 Table S10. (E)-2-Nonenal, octanoic acid, ethyl octanoate, nonanoic acid, and dihydro-5-pentyl-2(3H)-furanone were found in all the WW samples. When comparing all WW samples, the highest content of 2,5-dimethylpyrazine, 1-heptanol, 1-octen-3-ol, heptanoic acid, phenethyl alcohol, and dodecanoic acid were found in DS8472-5 blue WW fermented with the LUHS29 strain. The highest content of (E,E)-2,4-nonadienal, (E,E)-2,4-decadienal, 4-ethyl-3-nonen-5-yne, and trans-4,5-epoxy-(E)-2-decenal were found in ‘Sarta’ WW fermented with the LUHS245 strain. 4-methylhexanoic acid and ethylhydrocinnamate were found only in DS8472-5 blue WW fermented with the LUHS245 strain. The highest content of benzaldehyde was found in DS8526-2 purple WW. Blue WW DS8472-5 fermented with the LUHS245 strain showed the highest 2-nonanone and 4-ethylphenol content, and the nonfermented blue WW showed the highest content of nonanal. Found in only two samples was 4-methyl-1-(pent-4-en-1-yl)-2,3-diazabicyclo [2.2.1] hept-2-ene, and the highest content of this VC in nontreated blue WW was established.

**Figure 5:**
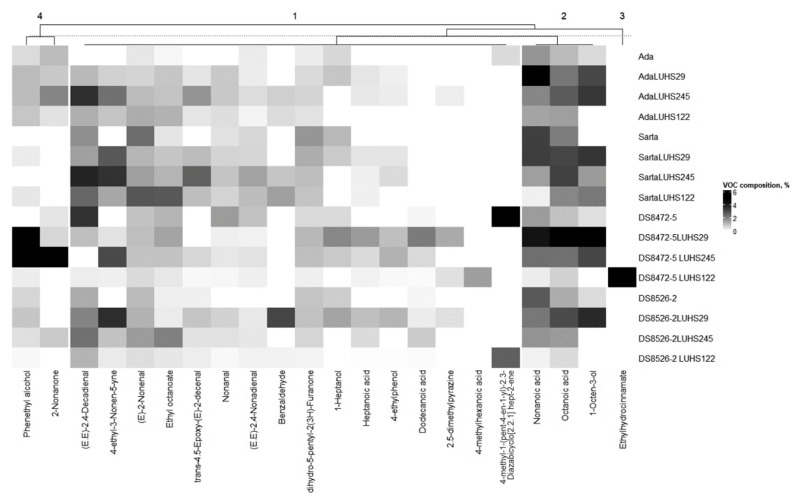
Volatile compounds that are <1% in wheat wholemeal samples (LUHS29—fermented with *Pediococcus acidilactici* LUHS29 strain; LUHS245—fermented with *Liquorilactobacillus uvarum* LUHS245 strain; LUHS122—fermented with *Lactiplantibacillus plantarum* LUHS122 strain. Data are represented as means (*n* = 3)).

VCs that were <1% in the WW samples are shown in Figure 5, and the detailed VC profile is given in Supplementary File S5 Table S11. Further, 3-methylbutanoic acid, 2-methylbutanoic acid, (Z)-3-hexadecene, butyl caprylate and propanoic acid, and 2-methyl-, 1-(1,1-dimethylethyl)-2-methyl-1,3-propanediyl ester were found in only purple WW fermented with the LUHS122 strain; heptanal was only found in nontreated ‘Sarta’; and oct-3-en-2-one, (Z)-2-nonen-4-yn-1-ol, and hexylbutyrate were found only in ‘Sarta’ WW fermented with LUHS122. Undecane was identified in nontreated blue WW, and 2-(ethylthio)propanoic acid ethyl ester; sorbic acid vinyl ester; and benzothiazole were only found in DS8472-5 blue WW fermented with the LUHS122 strain. It was found that 2,4,7,9-Tetramethyl-5-decyn-4,7-diol; 2,6,10-trimethyldodecane; 2,4-bis(1,1-dimethylethyl)phenol; pentadecane; and hexadecane were more typical for the colored WW samples (nontreated and fermented ones).

During the WW fermentation, many factors have an influence on VC formation, such as protein hydrolysis due to the cereal proteases at low pH, release of phenolic compounds, synthesis of flavor precursors, etc. [50]. LAB based sourdough has a more diverse VC profile in comparison with yeast-fermented doughs [85], and the most abundant VCs are carboxylic acids, esters, alcohols, ketones, aldehydes, and heterocycles [86]. It should be mentioned that the dominant VC produced by various processes of sourdough fermentation are often reported differently [82,86]. It was reported that the LAB fermentation can also mask bitterness due to the increased bran content in WW in comparison with refined flour, and these changes are related to the degradation of phenolic acids and aldehydes [87]. Sourdough fermentation can also increase the fruitiness [88]. Our results showed that the wheat variety, LAB strain used for fermentation, and their interaction are significant factors on the content of most of the identified VC compounds in the WW samples (Supplementary File S5 Table S12). Finally, according to the obtained results, a desirable VC profile of the wheat sourdoughs could be designed by selecting the appropriate wheat variety as well as LAB strains for its fermentation.

### 3.6. Changes in the Mycotoxin Concentration in Wheat Wholemeal during Fermentation

The mycotoxin concentration in the WW samples is shown in Table 6. In ‘Ada’ samples (nontreated and fermented) and nonfermented and LUHS122 strain fermented ‘Sarta’ samples, all the tested mycotoxin concentrations were below the detection limits. However, in ‘Sarta’ samples fermented with the LUHS29 and LUHS245 strain, the deoxynivalenol (DON) concentration was, on average, 19.5 μg/kg. Also, DON was established in all the tested DS8472-5 blue and DS8526-2 purple WW, with the highest DON concentration in samples fermented with LUHS29 (163.0 and 392.3 μg/kg, respectively). In DS8472-5 blue WW samples, after fermentation with all the tested LAB strains, the DON concentration was increased in comparison with the nontreated samples (in samples fermented with LUHS29 by 69.1%, in samples fermented with LUHS245 by 39.0%, and in samples fermented with LUHS122 by 20.3%). Different tendencies of the DON concentration in DS8526-2 purple WW samples were established, and after fermentation with LUHS29 and LUHS245, an increase in DON was found (by 43.0% and 18.0%, respectively); however, after fermentation with LUHS122, a 17.6% lower concentration of DON was established in the WW samples. It was reported that the anti-fungal activity of sourdough LAB is mainly attributed to the inhibitory metabolites produced during fermentation [89] and the synergistic interactions of the LAB metabolites with dough or microbial derived compounds, and this inhibitory effect may even change depending on the type of flour used [90,91]. However, the reduction of mycotoxins by LAB mainly depends on the binding of the toxin to the bacterial cell wall or, more rarely, due to the metabolism of the toxin [92]. Despite the fact that the main role in the removal of mycotoxins is attributed to the bacterial cell-wall components, [93] it was reported that the organic acids and antifungal ingredients such as phenyllactic acid, hydroxyphenyllactic acid, and indole lactic acid produced by the LAB also have anti-aflatoxigenic effects. To summarize, differences in the structure of the bacterial cell wall, the initial concentration of the toxin, the bacterial population, the incubation conditions, the type of the LAB, and probably the types and the amounts of the LAB metabolites are important in this effect [93,94]. Finally, it could be suggested that by changing the duration of the fermentation, moisture content of the fermentable substrate, etc., the results of the mycotoxin concentration in fermented WW could vary. Further research is needed to evaluate the influence of technological parameters on mycotoxin degradation in WW samples.

### 3.7. The Limitations and Perspectives of the Current Study

Taking into consideration that the functional and/or higher value food market is growing, the new data on the colored wheat chemical composition and its possible changes during fermentation are very important. Fermentation is the main process in bread as well as in cereal beverages preparation, and this study showed that despite all the tested WW and LAB strains being suitable for WW treatment, most of the parameters were related to the cereal variety, LAB strain used, and the interaction of these factors. From this point of view, further studies are needed to evaluate more parameters (i.e., enzyme activities, dietary fibre compounds, amino acids, etc.) and to optimize the fermentation process. Also, it would be very prospective to analyze the new breed lines cereal grains, which growth was performed at the different conditions, because the same conditions could have a different effect on different cereal breed lines grain chemical as well as technological characteristics. As a perspective, the application of fermented WW could be promising for various food products and feed preparation. However, taking into consideration the different technological requirements for different food materials, to produce good sensory properties possessing and safe food products, an optimization process (of the WW fermentation) could be performed specifically, just to obtain desirable properties for specific products.

## 4. Conclusions

All the fermented WW showed >8.0 log_10_ CFU/g of LAB count, and the type of LAB was a significant factor in the WW acidity parameters. Phenylethylamine was the predominant BA in WW, and the wheat variety (WV), the type of LAB, and their interaction were significant factors in the BA formation. Despite the fact that some differences in trace element concentrations in WW were obtained, in most of the cases fermentation was not a significant factor in their content. The main FAs in WW were palmitic acid, all-*cis*,*trans*-octadecenoic acid, linoleic acid. Fermented WW showed a more diverse VC profile; however, the influence of fermentation on deoxynivalenol in WW was varied. Finally, further studies are needed to indicate technological parameters that would be the most effective for each WV, including the lowest BA formation and mycotoxin degradation.

## Figures and Tables

**Table 1:** Acidity, chromaticity, and microbiological characteristics of the wheat wholemeal samples.

Wheat Samples	pH	TTA, °N	Color Coordinates, NBS			LAB Count, log_10_ CFU/g
			L*	a*	b*	
	**Traditional Wheat Variety**					
‘Ada’	6.68 ± 0.03 ^d,D^	1.30 ± 0.02 ^a,C^	52.8 ± 1.21 ^b,C^	5.79 ± 0.11 ^c,D^	16.6 ± 0.13 ^c,D^	4.41 ± 0.11 ^a,B^
‘Ada’_LUHS29_	4.29 ± 0.02 ^c,C^	4.90 ± 0.08 ^b,B^	42.0 ± 1.10 ^a,C^	4.27 ± 0.10 ^a,C^	8.88 ± 0.11 ^a,C^	8.52 ± 0.15 ^b,C^
‘Ada’_LUHS245_	4.21 ± 0.01 ^b,C^	4.80 ± 0.09 ^b,A^	50.6 ± 1.50 ^b,C^	5.46 ± 0.16 ^b,B^	15.7 ± 0.21 ^b,D^	8.61 ± 0.14 ^b,A^
‘Ada’_LUHS122_	3.99 ± 0.02 ^a,B^	5.10 ± 0.15 ^c,A^	51.9 ± 0.97 ^b,D^	5.79 ± 0.13 ^c,D^	16.8 ± 0.19 ^c,C^	8.72 ± 0.18 ^b,A^
	**Waxy Wheat Variety**					
‘Sarta’	6.42 ± 0.03 ^c,C^	1.00 ± 0.03 ^a,A^	40.9 ± 1.19 ^a,A^	4.43 ± 0.09 ^c,B^	8.10 ± 0.20 ^a,A^	4.33 ± 0.09 ^a,A^
‘Sarta’_LUHS29_	4.19 ± 0.02 ^b,B^	4.50 ± 0.09 ^b,A^	44.9 ± 0.94 ^b,D^	3.85 ± 0.07 ^a,b,A^	9.59 ± 0.14 ^b,D^	8.11 ± 0.21 ^b,A^
‘Sarta’_LUHS245_	3.84 ± 0.01 ^a,A^	5.50 ± 0.16 ^c,C^	46.7 ± 1.17 ^c,B^	3.72 ± 0.11 ^a,A^	9.89 ± 0.11 ^c,B^	8.37 ± 0.18 ^b,A^
‘Sarta’_LUHS122_	3.81 ± 0.02 ^a,A^	5.70 ± 0.12 ^c,B,C^	48.9 ± 1.41 ^d,B^	3.94 ± 0.08 ^b,A^	14.3 ± 0.13 ^d,B^	8.64 ± 0.16 ^b,A^
	**Blue Wheat—New Breed** **Line**					
DS8472-5	6.09 ± 0.02 ^d,A^	1.0 ± 0.02 ^a,A^	47.6 ± 0.32 ^b,B^	3.03 ± 0.07 ^c,A^	11.3 ± 0.09 ^b,C^	4.30 ± 0.06 ^a,A^
DS8472-5_LUHS29_	4.07 ± 0.01 ^b,A^	5.40 ± 0.08 ^c,C^	40.9 ± 0.27 ^a,B^	4.08 ± 0.06 ^b,B^	6.12 ± 0.14 ^a,B^	8.31 ± 0.12 ^bA,B^
DS8472-5_LUHS245_	4.15 ± 0.02 ^c,B^	5.10 ± 0.12 ^b,B^	50.3 ± 1.18 ^c,C^	6.85 ± 0.16 ^d,C^	13.8 ± 0.21 ^c,C^	8.53 ± 0.20 ^b,c,A^
DS8472-5_LUHS122_	3.94 ± 0.03 ^a,B^	5.80 ± 0.04 ^d,C^	50.6 ± 0.34 ^c,C,D^	4.76 ± 0.07 ^c,C^	14.0 ± 0.32 ^c,B^	8.92 ± 0.21 ^c,A^
	**Purple Wheat—New Breed** **Line**					
DS8526-2	6.18 ± 0.02 ^d,B^	1.20 ± 0.03 ^a,B^	46.1 ± 1.32 ^c,B^	5.12 ± 0.11 ^c,C^	9.52 ± 0.17 ^c,B^	4.53 ± 0.09 ^a,B^
DS8526-2_LUHS29_	4.24 ± 0.03 ^a,B,C^	5.00 ± 0.11 ^b,B^	38.6 ± 0.77 ^a,A^	4.25 ± 0.12 ^b,B,C^	5.42 ± 0.08 ^a,A^	8.31 ± 0.11 ^b,A,B^
DS8526-2_LUHS245_	4.86 ± 0.04 ^c,D^	5.90 ± 0.12 ^d,D^	40.3 ± 0.89 ^a,b,A^	3.58 ± 0.10 ^a,A^	5.29 ± 0.12 ^a,A^	8.45 ± 0.17 ^b,c,A^
DS8526-2_LUHS122_	4.67 ± 0.02 ^b,C^	5.60 ± 0.14 ^c,B^	39.8 ± 1.14 ^a,A^	4.10 ± 0.09 ^b,B^	5.86 ± 0.14 ^b,A^	8.76 ± 0.09 ^c,A^

TTA—total titratable acidity; L*—lightness; a*—redness (−a* greenness); b*—yellowness (−b* blueness); LAB—lactic acid bacteria; CFU—colony forming units; LUHS29—fermented with *Pediococcus acidilactici* LUHS29 strain; LUHS245—fermented with *Liquorilactobacillus uvarum* LUHS245 strain; LUHS122—fermented with *Lactiplantibacillus plantarum* LUHS122 strain. The data of physico-chemical parameters are represented as means (*n* = 3) ± SE. The data of lactic acid bacteria count are represented as means (*n* = 5) ± SE. ^a–d^ Means with different letters in the column are significantly different between the same variety of the non-treated and fermented samples (*p* ≤ 0.05). ^A–D^ Means with different letters in the column are significantly different between the different variety of samples (between non-treated and between the same LAB strain treated samples) (*p* ≤ 0.05).

**Table 2:** Biogenic amine concentration (mg/kg) in wheat wholemeal samples.

Wheat Samples	Biogenic Amine, mg/kg							
	TRY	PHE	PUT	CAD	HIS	TYR	SPRMD	SPRM
	**Traditional Wheat Variety**							
‘Ada’	nd	72.8 ± 5.4 ^b,C^	35.6 ± 2.9 ^b,A^	nd	nd	nd	23.4 ± 2.2 ^a,A^	nd
‘Ada’_LUHS29_	nd	56.9 ± 3.9 ^a,b,A^	20.3 ± 1.8 ^a,A^	nd	nd	nd	36.1 ± 3.3 ^b,B^	nd
‘Ada’_LUHS245_	nd	50.3 ± 3.4 ^a,B^	33.1 ± 2.5 ^b,B^	102.3 ± 6.9 ^a,B^	nd	nd	32.6 ± 3.8 ^b,A^	nd
‘Ada’_LUHS122_	nd	64.1 ± 4.7 ^b,B,C^	98.1 ± 6.3 ^c,B^	205.3 ± 9.8 ^a,B^	35.2 ± 2.6 ^A^	nd	nd	nd
	**Waxy Wheat Variety**							
‘Sarta’	nd	68.8 ± 5.0 ^b,B^	32.6 ± 2.6 ^b,A^	nd	nd	nd	nd	nd
‘Sarta’_LUHS29_	nd	68.6 ± 5.2 ^b,B^	28.9 ± 2.1 ^a,b,B^	nd	nd	nd	nd	nd
‘Sarta’_LUHS245_	nd	46.9 ± 3.1 ^a,B^	26.7 ± 1.9 ^a,A^	nd	nd	nd	31.9 ± 3.4 ^A^	nd
‘Sarta’_LUHS122_	nd	55.6 ± 3.6 ^a,A^	26.6 ± 2.1 ^a,A^	nd	77.4 ± 5.4 ^B^	nd	nd	nd
	**Blue Wheat—New Breed** **Line**							
DS8472-5	nd	57.2 ± 3.9 ^a,b,A^	nd	nd	nd	nd	nd	nd
DS8472-5_LUHS29_	nd	53.6 ± 3.5 ^a,A^	nd	nd	nd	nd	26.7 ± 3.2 ^a,A^	nd
DS8472-5_LUHS245_	nd	62.2 ± 4.4 ^b,C^	nd	nd	nd	nd	36.5 ± 3.8 ^b,B^	nd
DS8472-5_LUHS122_	nd	68.7 ± 5.1 ^b,C^	nd	88.0 ± 5.9 ^A^	nd	nd	31.9 ± 4.1 ^b,A^	nd
	**Purple Wheat—New Breed** **Line**							
DS8526-2	nd	58.0 ± 4.3 ^b,A^	nd	nd	nd	nd	21.4 ± 2.3 ^a,A^	nd
DS8526-2_LUHS29_	nd	70.4 ± 5.3 ^c,B^	nd	nd	nd	nd	30.7 ± 3.5 ^c,A^	nd
DS8526-2_LUHS245_	nd	26.4 ± 2.4 ^a,A^	26.8 ± 2.7 ^A^	84.0 ± 5.8 ^a,A^	nd	nd	27.2 ± 3.1 ^b,c,A^	nd
DS8526-2_LUHS122_	nd	51.9 ± 3.2 ^b,A^	nd	99.9 ± 6.5 ^b,A^	nd	76.0 ± 5.4	26.3 ± 2.1 ^b,A^	nd

LUHS29—fermented with *Pediococcus acidilactici* LUHS29 strain; LUHS245—fermented with *Liquorilactobacillus uvarum* LUHS245 strain; LUHS122—fermented with *Lactiplantibacillus plantarum* LUHS122 strain.; TRY—tryptamine; PUTR—putrescine; CAD—cadaverine; HIS—histamine; TYR—tyramine; SPRMD—spermidine; SPRM—spermine; nd—not found. Data are represented as means (*n* = 3) ± SE. ^a–c^ Means with different letters in the column are significantly different between the same variety of non-treated and fermented samples (*p* ≤ 0.05). ^A–C^ Means with different letters in the column are significantly different between the different variety of samples (between non-treated and between the same LAB strain treated samples) (*p* ≤ 0.05).

**Table 3:** Macroelement concentrations in wheat wholemeal samples.

Wheat Samples	Macroelements, d.m.			
	Na, g/100 g	Mg, g/kg	K, g/kg	Ca, g/kg
	**Traditional Wheat Variety**			
‘Ada’	<0.002	0.662 ± 0.066 ^a,B^	1.98 ± 0.194 ^a,B^	0.229 ± 0.023 ^a,C^
‘Ada’_LUHS29_	0.010 ± 0.001 ^a,B^	0.601 ± 0.060 ^a,B^	1.91 ± 0.187 ^a,A^	0.192 ± 0.019 ^a,B^
‘Ada’_LUHS245_	0.007 ± 0.002 ^a,B^	0.551 ± 0.055 ^a,A^	1.74 ± 0.170 ^a,A,B^	0.197 ± 0.018 ^a,B^
‘Ada’_LUHS122_	0.007 ± 0.002 ^a,A^	0.578 ± 0.058 ^a,D^	1.79 ± 0.175 ^a,B^	0.197 ± 0.020 ^a,C^
	**Waxy Wheat Variety**			
‘Sarta’	<0.002	0.555 ± 0.056 ^c,B^	1.98 ± 0.194 ^c,B^	0.305 ± 0.031 ^c,D^
‘Sarta’_LUHS29_	0.011 ± 0.002 ^a,B^	0.607 ± 0.061 ^c,B^	1.82 ± 0.178 ^b,c,A^	0.293 ± 0.029 ^c,C^
‘Sarta’_LUHS245_	0.009 ± 0.001 ^a,B^	0.479 ± 0.048 ^b,A^	1.52 ± 0.152 ^b,A^	0.225 ± 0.025 ^b,B^
‘Sarta’_LUHS122_	0.011 ± 0.002 ^a,A^	0.373 ± 0.037 ^a,B^	1.15 ± 0.113 ^a,A^	0.160 ± 0.015 ^a,B^
	**Blue Wheat—New Breed** **Line**			
DS8472-5	<0.002	0.401 ± 0.040 ^a,A^	1.45 ± 0.150 ^a,A^	0.160 ± 0.014 ^c,B^
DS8472-5 _LUHS29_	0.008 ± 0.001 ^a,B^	0.445 ± 0.040 ^a,A^	1.96 ± 0.200 ^b,A^	0.148 ± 0.015 ^a,b,A^
DS8472-5 _LUHS245_	0.009 ± 0.001 ^a,B^	0.431 ± 0.043 ^a,A^	1.71 ± 0.170 ^a,b,A,B^	0.126 ± 0.013 ^a,A^
DS8472-5 _LUHS122_	0.007 ± 0.002 ^a,A^	0.456 ± 0.046 ^a,C^	1.76 ± 0.180 ^a,b,B^	0.147 ± 0.015 ^a,b,A,B^
	**Purple Wheat—New Breed** **Line**			
DS8526-2	<0.002	0.518 ± 0.025 ^c,B^	1.93 ± 0.190 ^b,B^	0.146 ± 0.015 ^a,A^
DS8526-2 _LUHS29_	0.005 ± 0.001 ^a,A^	0.376 ± 0.038 ^b,A^	1.96 ± 0.160 ^b,A^	0.124 ± 0.012 ^a,A^
DS8526-2 _LUHS245_	0.004 ± 0.001 ^a,A^	0.500 ± 0.050 ^c,A^	2.00 ± 0.200 ^b,B^	0.129 ± 0.013 ^a,A^
DS8526-2 _LUHS122_	0.006 ± 0.003 ^a,A^	0.292 ± 0.029 ^a,A^	1.11 ± 0.110 ^a,A^	0.134 ± 0.011 ^a,A^

LUHS29—fermented with *Pediococcus acidilactici* LUHS29 strain; LUHS245—fermented with *Liquorilactobacillus uvarum* LUHS245 strain; LUHS122—fermented with *Lactiplantibacillus plantarum* LUHS122 strain. Data are represented as means (*n* = 3) ± SE. ^a–c^ Means with different letters in the column are significantly different between the same variety of non-treated and fermented samples (*p* ≤ 0.05). ^A–D^ Means with different letters in the column are significantly different between the different variety of samples (between non-treated and between the same LAB strain treated samples) (*p* ≤ 0.05).

**Table 4:** Essential microelement concentrations in wheat wholemeal samples.

Wheat Samples	Essential Microelements, d.m.							
	Cr, mg/100 g	Mn, mg/kg	Fe, mg/kg	Co, mg/kg	Ni, mg/kg	Cu, mg/kg	Zn, mg/kg	Se, mg/kg
**Traditional Wheat Variety**								
‘Ada’	<0.010	8.21 ± 0.820 ^c,A^	22.9 ± 2.60 ^a,B^	<0.010	<0.500	1.85 ± 0.190 ^a,D^	6.98 ± 0.70 ^b,A^	<0.200
‘Ada’_LUHS29_	<0.010	9.00 ± 0.900 ^c,A^	28.3 ± 2.80 ^a,C^	<0.010	<0.500	1.51 ± 0.150 ^a,C^	7.53 ± 0.75 ^b,B^	<0.200
‘Ada’_LUHS245_	<0.010	5.83 ± 0.580 ^a,A^	23.2 ± 2.28 ^a,B^	<0.010	<0.500	1.59 ± 0.160 ^a,C^	5.51 ± 0.55 ^a,A^	<0.200
‘Ada’_LUHS122_	0.014 ± 0.001	7.81 ± 0.780 ^b,A^	38.7 ± 3.90 ^b,C^	<0.010	<0.500	1.61 ± 0.157 ^a,B^	7.05 ± 0.71 ^b,B^	<0.200
	**Waxy Wheat Variety**							
‘Sarta’	0.047 ± 0.001 ^a,B^	10.4 ± 1.00 ^a,b,A^	14.0 ± 1.40 ^b,A^	<0.010	<0.500	1.24 ± 0.120 ^b,C^	6.95 ± 0.70 ^c,A^	<0.200
‘Sarta’_LUHS29_	0.052 ± 0.005 ^a^	13.4 ± 1.30 ^c,C^	15.4 ± 1.50 ^b,B^	<0.010	<0.500	1.32 ± 0.130 ^b,C^	7.14 ± 0.71 ^c,A^	<0.200
‘Sarta’_LUHS245_	<0.010	12.1 ± 1.20 ^b,c,B,C^	22.0 ± 2.20 ^c,B^	<0.010	<0.500	1.27 ± 0.128 ^b,C^	5.23 ± 0.52 ^b,A^	<0.200
‘Sarta’_LUHS122_	<0.010	8.25 ± 0.820 ^a,A^	5.48 ± 0.550 ^a,A^	<0.010	<0.500	0.830 ± 0.083 ^a,A^	3.61 ± 0.36 ^a,A^	<0.200
	**Blue Wheat—New Breed** **Line**							
DS8472-5	0.015 ± 0.002 ^A^	10.7 ± 1.00 ^a,A^	14.9 ± 1.50 ^a,A^	<0.010	<0.500	0.763 ± 0.076 ^a,A^	7.25 ± 0.73 ^a,A^	<0.200
DS8472-5_LUHS29_	<0.010	13.3 ± 1.30 ^b,C^	16.6 ± 1.70 ^a,B^	<0.010	<0.500	0.857 ± 0.086 ^a,B^	7.05 ± 0.71 ^a,A^	<0.200
DS8472-5_LUHS245_	<0.010	10.9 ± 1.10 ^a,B^	13.7 ± 1.40 ^a,A^	<0.010	<0.500	0.695 ± 0.070 ^a,A^	6.64 ± 0.66 ^a,A,B^	<0.200
DS8472-5_LUHS122_	<0.010	14.0 ± 1.40 ^b,B^	15.6 ± 1.60 ^a,B^	<0.010	<0.500	0.850 ± 0.085 ^a,A^	6.70 ± 0.67 ^a,B^	<0.200
	**Purple Wheat—New Breed** **Line**							
DS8526-2	<0.010	15.3 ± 1.50 ^c,B^	16.5 ± 1.70 ^b,A^	<0.010	<0.500	0.913 ± 0.091 ^b,B^	7.88 ± 0.79 ^b,A^	<0.200
DS8526-2_LUHS29_	<0.010	11.1 ± 1.10 ^b,B^	10.3 ± 1.10 ^a,A^	<0.010	<0.500	0.692 ± 0.069 ^a,A^	6.03 ± 0.60 ^a,A^	<0.200
DS8526-2_LUHS245_	<0.010	13.8 ± 1.40 ^c,C^	15.9 ± 1.60 ^b,A^	<0.010	<0.500	0.848 ± 0.085 ^a,b,A^	7.86 ± 0.79 ^b,B^	<0.200
DS8526-2_LUHS122_	<0.010	8.71 ± 0.870 ^a,A^	17.6 ± 1.80 ^b,B^	<0.010	<0.500	0.728 ± 0.073 ^a,A^	6.11 ± 0.61 ^a,B^	<0.200

LUHS29—fermented with *Pediococcus acidilactici* LUHS29 strain; LUHS245—fermented with *Liquorilactobacillus uvarum* LUHS245 strain; LUHS122—fermented with *Lactiplantibacillus plantarum* LUHS122 strain. Data are represented as means (*n* = 3) ± SE. ^a–c^ Means with different letters in the column are significantly different between the same variety of non-treated and fermented samples (*p* ≤ 0.05). ^A–D^ Means with different letters in the column are significantly different between the different variety of samples (between non-treated and between the same LAB strain treated samples) (*p* ≤ 0.05).

**Table 5:** Non-essential microelement concentrations in wheat wholemeal samples.

Wheat Samples	Non-Essential Macroelements, d.m.													
	As, mg/100 g	V, mg/kg	Rb, mg/kg	Sr, mg/kg	Mo, mg/kg	Ag, mg/kg	Sb, mg/kg	Cs, mg/kg	TI, mg/kg	Cd, mg/kg	Ba, mg/kg	Pb, mg/kg	Al, mg/kg	Li, mg/kg
	**Traditional Wheat Variety**													
‘Ada’	<0.050	<2.00	<1.00	1.00 ± 0.100 ^a,A^	<0.500	<2.00	<0.500	<2.00	<2.00	0.038 ± 0.004 ^b,C^	2.71 ± 0.270 ^a,A^	0.037 ± 0.002 ^a^	<5.00	<0.050
‘Ada’_LUHS29_	<0.050	<2.00	<1.00	0.949 ± 0.095 ^a,A^	<0.500	<2.00	<0.500	<2.00	<2.00	0.029 ± 0.003 ^a,B^	2.36 ± 0.240 ^a,A^	0.032 ± 0.003 ^a^	<5.00	<0.050
‘Ada’_LUHS245_	0.009 ± 0.001 ^A^	<2.00	<1.00	0.854 ± 0.085 ^a,A^	<0.500	<2.00	<0.500	<2.00	<2.00	0.037 ± 0.004 ^b,C^	2.27 ± 0.229 ^a,A^	0.038 ± 0.007 ^a^	<5.00	<0.050
‘Ada’_LUHS122_	<0.050	<2.00	<1.00	0.909 ± 0.091 ^a,b,A^	<0.500	<2.00	<0.500	<2.00	<2.00	0.033 ± 0.003 ^a,b,D^	2.30 ± 0.230 ^a,A^	0.034 ± 0.006 ^a^	<5.00	<0.050
	**Waxy Wheat Variety**													
‘Sarta’	0.007 ± 0.003 ^a,A^	<2.00	<1.00	1.22 ± 0.11 ^c,A^	<0.500	<2.00	<0.500	<2.00	<2.00	0.027 ± 0.003 ^a,B^	2.30 ± 0.225 ^a,A^	<0.010	<5.00	<0.050
‘Sarta’_LUHS29_	0.011 ± 0.002 ^a,A^	<2.00	<1.00	1.20 ± 0.12 ^c,A,B^	<0.500	<2.00	<0.500	<2.00	<2.00	0.026 ± 0.004 ^a,B^	2.38 ± 0.240 ^a,A^	<0.010	<5.00	<0.050
‘Sarta’_LUHS245_	<0.050	<2.00	<1.00	1.02 ± 0.10 ^b,A^	<0.500	<2.00	<0.500	<2.00	<2.00	0.028 ± 0.003 ^a,B^	2.11 ± 0.210 ^a,A^	<0.010	<5.00	<0.050
‘Sarta’_LUHS122_	0.013 ± 0.003 ^a,A^	<2.00	<1.00	0.719 ± 0.072 ^a,A^	<0.500	<2.00	<0.500	<2.00	<2.00	0.022 ± 0.003 ^a,C^	2.13 ± 0.209 ^a,A^	<0.010	<5.00	<0.050
	**Blue Wheat—New Breed** **Line**													
DS8472-5	<0.050	<2.00	<1.00	1.46 ± 0.15 ^a,B^	<0.500	<2.00	<0.500	<2.00	<2.00	0.009 ± 0.001 ^a,A^	<2.00	<0.010	<5.00	<0.050
DS8472-5_LUHS29_	<0.050	<2.00	<1.00	1.61 ± 0.16 ^a,B^	<0.500	<2.00	<0.500	<2.00	<2.00	0.010 ± 0.002 ^a,A^	<2.00	<0.010	<5.00	<0.050
DS8472-5_LUHS245_	<0.050	<2.00	<1.00	1.44 ± 0.14 ^a,B^	<0.500	<2.00	<0.500	<2.00	<2.00	0.009 ± 0.001 ^a,A^	<2.00	<0.010	<5.00	<0.050
DS8472-5_LUHS122_	<0.050	<2.00	<1.00	1.67 ± 0.17 ^a,D^	<0.500	<2.00	<0.500	<2.00	<2.00	0.012 ± 0.002 ^a,B^	<2.00	<0.010	<5.00	<0.050
	**Purple Wheat—New Breed** **Line**													
DS8526-2	<0.050	<2.00	<1.00	1.90 ± 0.19 ^b,B^	<0.500	<2.00	<0.500	<2.00	<2.00	0.009 ± 0.001 ^a,A^	<2.00	<0.010	<5.00	<0.050
DS8526-2_LUHS29_	<0.050	<2.00	<1.00	1.45 ± 0.15 ^a,B^	<0.500	<2.00	<0.500	<2.00	<2.00	0.008 ± 0.001 ^a,A^	<2.00	<0.010	<5.00	<0.050
DS8526-2_LUHS245_	<0.050	<2.00	<1.00	1.84 ± 0.18 ^b,C^	<0.500	<2.00	<0.500	<2.00	<2.00	0.009 ± 0.001 ^a,A^	<2.00	<0.010	<5.00	<0.050
DS8526-2_LUHS122_	<0.050	<2.00	<1.00	1.32 ± 0.13 ^a,C^	<0.500	<2.00	<0.500	<2.00	<2.00	0.008 ± 0.001 ^a,A^	<2.00	<0.010	<5.00	<0.050

LUHS29—fermented with *Pediococcus acidilactici* LUHS29 strain; LUHS245—fermented with *Liquorilactobacillus uvarum* LUHS245 strain; LUHS122—fermented with *Lactiplantibacillus plantarum* LUHS122 strain. Data are represented as means (*n* = 3) ± SE. ^a–c^ Means with different letters in the column are significantly different between the same variety of non-treated and fermented samples (*p* ≤ 0.05). ^A^^–^^D^ Means with different letters in the column are significantly different between the different variety of samples (between non-treated and between the same LAB strain treated samples) (*p* ≤ 0.05).

**Table 6:** Mycotoxin concentration in wheat wholemeal samples.

Wheat Samples	Mycotoxin Concentration, μg/kg						
	DON	HT2	T2	FB1	FB2	ZEA	OTA
	**Traditional Wheat Variety**						
‘Ada’	<15	<1.5	<1.5	<15	<15	<3	<1.5
‘Ada’_LUHS29_							
‘Ada’_LUHS245_							
‘Ada’_LUHS122_							
	**Waxy Wheat Variety**						
‘Sarta’	<15	<1.5	<1.5	<15	<15	<3	<1.5
‘Sarta’_LUHS29_	21.5 ± 2.1 ^a,B^						
‘Sarta’_LUHS245_	17.4 ± 1.8 ^a,B^						
‘Sarta’_LUHS122_	<15						
	**Blue Wheat—New Breed** **Line**						
DS8472-5	96.4 ± 4.2 ^a,A^	<1.5	<1.5	<15	<15	<3	<1.5
DS8472-5_LUHS29_	163.0 ± 7.8 ^d,A^						
DS8472-5_LUHS245_	134.0 ± 6.5 ^c,A^						
DS8472-5_LUHS122_	116.0 ± 9.2 ^b,A^						
	**Purple Wheat—New Breed** **Line**						
DS8526-2	274.4 ± 9.8 ^d,B^	<1.5	<1.5	<15	<15	<3	<1.5
DS8526-2_LUHS29_	392.3 ± 11.3 ^b,B^						
DS8526-2_LUHS245_	323.9 ± 14.5 ^c,B^						
DS8526-2_LUHS122_	226.0 ± 11.6 ^a,B^						

LUHS29—fermented with *Pediococcus acidilactici* LUHS29 strain; LUHS245—fermented with *Liquorilactobacillus uvarum* LUHS245 strain; LUHS122—fermented with *Lactiplantibacillus plantarum* LUHS122 strain.DON—deoxynivalenol; HT2—HT2 toxin; T2—T2 toxin; FB1—fumonisin B1; FB2—fumonisin B2; ZEA—zearalenone; OTA—ochratoxin A. Data are represented as means (*n* = 3) ± SE. ^a–d^ Means with different letters in the column are significantly different between the same variety of non-treated and fermented samples (*p* ≤ 0.05). ^A,B^ Means with different letters in the column are significantly different between the different variety of samples (between non-treated and between the same LAB strain treated samples) (*p* ≤ 0.05).

## Data Availability

Not applicable.

## Supplementary Materials

The following supporting information can be downloaded at: https://www.mdpi.com/article/10.3390/biology11070966/s1, Supplementary File S1. Instrumental parameters for the HPLC-MS/MS analysis of mycotoxins: Table S1. HPLC parameters. Table S2. Analyzed compound retention time, precursor and product *m*/*z.* Supplementary File S2. Statistical data of the WW acidity, chromaticity, microbiological characteristics: Table S3. The influence of the wheat variety and type of LAB on the acidity, chromaticity, and microbiological characteristics of the WW samples. Table S4. Pearson correlations between the WW acidity, chromaticity, and microbiological characteristics. Supplementary File S3. Statistical data of the biogenic amines in WW samples: Table S5. The influence of the wheat variety and type of LAB on the biogenic amine content in WW samples. Table S6. Pearson correlations between the WW acidity, chromaticity, and microbiological characteristics. Supplementary File S4. Table S7. Fatty acid profile of the wheat samples. Table S8. The influence of the wheat variety and type of LAB on the fatty acid content in WW samples. Supplementary File S5. Wheat sample volatile compounds: Table S9. Volatile compounds that are >5% in at least one wheat wholemeal sample. Table S10. Volatile compounds that are >1% and <5% in at least one wheat wholemeal sample. Table S11. Volatile compounds that are <1% in at least one wheat wholemeal sample. Table S12. The influence of the wheat variety and type of LAB on the acidity, chromaticity, and microbiological characteristics of the WW samples.
