# Supplementary material for: Comparison Study of Nontreated and Fermented Wheat Varieties ‘Ada’, ‘Sarta’, and New Breed Blue and Purple Wheat Lines Wholemeal Flour
Source: Biology (Basel). 2022 Jun 27;11(7):966. doi: 10.3390/biology11070966 (PMC9312326; doi:10.3390/biology11070966)
Supplement: Supplementary file 1 [file biology-11-00966-s001.zip › Supplementary File S1. Instrumental parameters for the HPLC-MS-MS analysis of mycotoxins.pdf]

Instrumental parameters for the HPLC-MS/MS analysis of mycotoxins

1) **HPLC parameters**

Phase A – 0.1% formic acid, 0,5mM ammonium acetate in water
Phase B - 0.1% formic acid, 0,5mM ammonium acetate in methanol

Table S1. HPLC parameters.

	Flow Gradient	
Time, min	A, %	B, %
0,0	85	15
1,0	85	15
4,5	2	98
5,2	2	98
6,5	90	10
7,5	90	10
8,5	85	15
10,5	85	15

2) **MS/MS parameters**

Method Duration (min): 10,5

Ion Source
- Ion Source Type: H-ESI
- Spray Voltage: Static
- Positive Ion (V): 3500
- Negative Ion (V): 2500
- Sheath Gas (Arb): 55
- Aux Gas (Arb): 25
- Sweep Gas (Arb): 5
- Ion Transfer Tube Temp (°C): 300
- Vaporizer Temp (°C): 350

SRM Scan:
- Use Cycle Time: True
- Cycle Time (sec): 0.4
- Use Calibrated RF Lens: True
- Q1 Resolution (FWHM): 0.7
- Q3 Resolution (FWHM): 1.2
- CID Gas (mTorr): 1.5

Table S2. Analysed compound retention time, precursor and product *m/z*.

Analysed compound retention time, precursor and product *m/z*					
Compound	Retention time, min	Mode	Precursor *m/z*	Product *m/z*	Collision energy, *V*
Aflatoxin B1	6,06	+	313	213	43
				241	37
				273	22
Deoxynivalenol	2,08	+	297	231	12
				249	11
Ochratoxin A	7,00	+	404	239	25
				358	13
Fumonisin B1	6,44	+	722	334	21
				352	40
Fumonisin B2	6,86	+	706	318	36
				336	38
T-2	6,75	+	489	327	23
				387	21
HT-2	6,49	+	442	215	10
				263	10
Zearalenone	6,99	-	317	273	18
				157	24
