# Supplementary material for: Comparison Study of Nontreated and Fermented Wheat Varieties ‘Ada’, ‘Sarta’, and New Breed Blue and Purple Wheat Lines Wholemeal Flour
Source: Biology (Basel). 2022 Jun 27;11(7):966. doi: 10.3390/biology11070966 (PMC9312326; doi:10.3390/biology11070966)
Supplement: Supplementary file 1 [file biology-11-00966-s001.zip › Supplementary File S2. Statistical data of the WW acidity, chromaticity, microbiological characteristics .pdf]

Table S3. The influence of the wheat variety and type of LAB on the acidity, chromaticity, and microbiological characteristics of the WW samples.

Source	Dependent Variable	p
Wheat variety	pH	0.797
	TTA	0.995
	L	0.507
	a	0.585
	b	0.126
	LAB count	0.705
The type of LAB	pH	**0.0001**
	TTA	**0.011**
	L	0.551
	a	0.669
	b	0.882
	LAB count	**0.0001**
Wheat variety * The type of LAB	pH	0.416
	TTA	1.000
	L	0.852
	a	**0.004**
	b	0.513
	LAB count	0.567

TTA – total titratable acidity; L* – lightness; a* – redness (−a* greenness); b* – yellowness (−b* blueness); LAB – lactic acid bacteria. The influence of analysed factors and their interaction is significant, when p ≤ 0.05. In Bold letters are marked significant values.

Table S4. Pearson correlations between the WW acidity, chromaticity, and microbiological characteristics.

		pH	TTA	L	a	b	LAB count
pH	r	1	**-0.422****	0.007	-0.002	-0.146	**-0.406****
	p		0.003	0.962	0.990	0.321	0.004
TTA	r	**-0.422****	1	**0.578****	0.073	**0.341***	0.195
	p	0.003		0.0001	0.624	0.018	0.184
L	r	0.007	**0.578****	1	0.031	**0.403****	-0.268
	p	0.962	0.0001		0.833	0.005	0.065
a	r	-0.002	0.073	0.031	1	-0.049	0.042
	p	0.990	0.624	0.833		0.743	0.776
b	r	-0.146	**0.341***	**0.403****	-0.049	1	-0.138
	p	0.321	0.018	0.005	0.743		0.351
LAB count	r	**-0.406****	0.195	-0.268	0.042	-0.138	1
	p	0.004	0.184	0.065	0.776	0.351	

r - Pearson Correlation; p – significance; TTA – total titratable acidity; L* – lightness; a* – redness (−a* greenness); b* – yellowness (−b* blueness); LAB – lactic acid bacteria; ** Correlation is significant at the 0.01 level (2-tailed). * Correlation is significant at the 0.05 level (2-tailed).

In Bold letters are marked significant values.
