# Supplementary material for: Comparison Study of Nontreated and Fermented Wheat Varieties ‘Ada’, ‘Sarta’, and New Breed Blue and Purple Wheat Lines Wholemeal Flour
Source: Biology (Basel). 2022 Jun 27;11(7):966. doi: 10.3390/biology11070966 (PMC9312326; doi:10.3390/biology11070966)
Supplement: Supplementary file 1 [file biology-11-00966-s001.zip › Supplementary File S3. Statistical data of the biogenic amines in WW samples.pdf]

Table S5. The influence of the wheat variety and type of LAB on the biogenic amines content in WW samples.

Source	Dependent Variable	p
Wheat variety	Phenylethylamine	0.0001
	Putrescine	0.0001
	Cadaverine	0.0001
	Histamine	0.006
	Tyramine	0.004
	Spermidine	0.0001
The type of LAB	Phenylethylamine	0.0001
	Putrescine	0.0001
	Cadaverine	0.0001
	Histamine	0.0001
	Tyramine	0.004
	Spermidine	0.0001
Wheat variety * The type of LAB	Phenylethylamine	0.0001
	Putrescine	0.0001
	Cadaverine	0.0001
	Histamine	0.0001
	Tyramine	0.0001
	Spermidine	0.0001

LAB – lactic acid bacteria. The influence of analysed factors and their interaction is significant, when $p \le 0.05$.

Table S6. Pearson correlations between the WW acidity, chromaticity, and microbiological characteristics.

		pH	TTA	LAB count	PHE	PUTR	CAD	HIS	TYR	SPRMD
pH	r	1	**-0.422****	**-0.406****	**0.389****	-0.064	-0.270	-0.130	0.036	-0.247
	p		0.003	0.004	0.006	0.664	0.064	0.380	0.808	0.090
TTA	r	**-0.422****	1	0.195	-0.272	0.029	0.262	0.057	0.222	0.153
	p	0.003		0.184	0.061	0.844	0.072	0.700	0.130	0.300
LAB count	r	**-0.406****	0.195	1	-0.230	-0.031	0.202	0.091	0.160	**0.422****
	p	0.004	0.184		0.116	0.833	0.168	0.536	0.276	0.003
PHE	r	**0.389****	-0.272	-0.230	1	0.053	-0.137	-0.069	-0.057	-0.365*
	p	0.006	0.061	0.116		0.720	0.353	0.642	0.701	0.011
PUTR	r	-0.064	0.029	-0.031	0.053	1	**0.639****	**0.434****	-0.171	**-0.322***
	p	0.664	0.844	0.833	0.720		0.0001	0.002	0.246	0.026
CAD	r	-0.270	0.262	0.202	-0.137	**0.639****	1	0.227	0.250	-0.132
	p	0.064	0.072	0.168	0.353	0.0001		0.120	0.086	0.372
HIS	r	-0.130	0.057	0.091	-0.069	**0.434****	0.227	1	-0.069	**-0.430****
	p	0.380	0.700	0.536	0.642	0.002	0.120		0.642	0.002
TYR	r	0.036	0.222	0.160	-0.057	-0.171	0.250	-0.069	1	0.104
	p	0.808	0.130	0.276	0.701	0.246	0.086	0.642		0.481
SPRMD	r	-0.247	0.153	**0.422****	**-0.365***	**-0.322***	-0.132	**-0.430****	0.104	1
	p	0.090	0.300	0.003	0.011	0.026	0.372	0.002	0.481	

r - Pearson Correlation; p – significance; TTA – total titratable acidity; LAB – lactic acid bacteria; PHE – phenylethylamine; PUTR – putrescine; CAD – cadaverine; HIS – histamine; TYR – tyramine; SPRMD – spermidine.
** Correlation is significant at the 0.01 level (2-tailed). * Correlation is significant at the 0.05 level (2-tailed).
In Bold letters are marked significant values.
