# Supplementary material for: Comparison Study of Nontreated and Fermented Wheat Varieties ‘Ada’, ‘Sarta’, and New Breed Blue and Purple Wheat Lines Wholemeal Flour
Source: Biology (Basel). 2022 Jun 27;11(7):966. doi: 10.3390/biology11070966 (PMC9312326; doi:10.3390/biology11070966)
Supplement: Supplementary file 1 [file biology-11-00966-s001.zip › Supplementary File S4. Fatty acids profile of the wheat samples.pdf]

Table S7. Fatty acids profile of wheat whole meal samples.

Wheat Samples	Fatty Acids Concentration, % from Total Fat Content																			
	C14:0	C16:0	C16:1	C17:0	C18:0	C18:1 cis-trans	C18:2	C18:3 γ	C18:3 α	C20:0	C20:1	C20:4	C22:0	C22:1	SFA	MUFA	PUFA	omega 3	omega 6	omega 9
**Traditional Wheat Variety**																				
'Ada'	2.93 ±0.01	28.4 ±0.1	0.771 ±0.002	nd	8.51 ±0.03	21.7 ±0.1	34.8 ±0.2	nd	2.11 ±0.01	nd	0.594 ±0.002	nd	nd	nd	39.95	23.12	36.93	2.11	34.82	23.12
'Ada'$_{\text{LUHS29}}$	0.236 ±0.001	23.7 ±0.1	nd	nd	5.76 ±0.02	16.9 ±0.1	50.7 ±0.2	nd	2.55 ±0.02	nd	nd	nd	nd	nd	29.74	16.94	53.31	2.56	50.76	16.94
'Ada'$_{\text{LUHS245}}$	0.430 ±0.003	22.1 ±0.1	0.228 ±0.002	nd	8.72 ±0.24	20.4 ±0.1	44.1 ±0.2	nd	3.09 ±0.1	0.125 ±0.002	0.617 ±0.003	nd	nd	nd	31.42	21.33	47.25	3.09	44.15	21.33
'Ada'$_{\text{LUHS122}}$	1.37 ±0.04	27.2 ±0.2	nd	nd	7.00 ±0.10	17.1 ±0.2	44.8 ±0.3	nd	2.07 ±0.1	nd	0.592 ±0.002	nd	nd	nd	39.95	23.12	36.93	2.11	34.82	23.12
**Waxy Wheat Variety**																				
'Sarta'	0.819 ±0.003	19.4 ±0.10	0.245 ±0.002	0.022 ±0.001	3.37 ±0.02	14.6 ±0.1	54.1 ±0.2	0.082 ±0.01	6.23 ±0.03	0.111 ±0.003	0.597 ±0.002	0.149 ±0.005	0.190 ±0.001	nd	23.88	15.46	60.66	6.23	54.43	15.46
'Sarta'$_{\text{LUHS29}}$	nd	19.1 ±0.1	nd	nd	2.25 ±0.02	12.8 ±0.1	59.2 ±0.2	nd	5.27 ±0.02	nd	1.34 ±0.01	nd	nd	nd	21.32	14.18	64.50	5.27	59.23	14.18
'Sarta'$_{\text{LUHS245}}$	nd	22.5 ±0.1	nd	nd	8.47 ±0.2	21.1 ±0.2	45.1 ±0.3	nd	2.84 ±0.02	nd	nd	nd	nd	nd	30.92	21.12	47.95	2.84	45.11	21.12
'Sarta'$_{\text{LUHS122}}$	nd	24.0 ±0.1	nd	nd	3.86 ±0.2	18.0 ±0.1	50.2 ±0.2	nd	2.94 ±0.01	nd	0.889 ±0.003	nd	nd	nd	27.87	18.90	53.23	2.94	50.29	18.90
**Blue Wheat - New Bred Line**																				
DS8472-5	nd	25.3 ±0.2	nd	nd	7.23 ±0.12	19.9 ±0.1	44.7 ±0.3	nd	2.73 ±0.01	nd	nd	nd	nd	nd	32.57	19.99	47.44	2.73	44.71	19.99
DS8472-5$_{\text{LUHS29}}$	nd	20.8 ±0.2	nd	nd	4.71 ±0.03	18.4 ±0.1	52.5 ±0.2	nd	2.93 ±0.04	nd	0.634 ±0.002	nd	nd	nd	25.49	19.08	55.43	2.93	52.50	19.08
DS8472-5$_{\text{LUHS245}}$	2.42 ±0.08	24.0 ±0.1	0.311 ±0.002	nd	5.52 ±0.16	18.3 ±0.2	45.9 ±0.3	nd	2.89 ±0.02	nd	0.533 ±0.003	nd	nd	nd	31.98	19.14	48.87	2.89	45.99	19.14
DS8472-5$_{\text{LUHS122}}$	nd	21.0 ±0.2	nd	nd	3.96 ±0.11	17.4 ±0.1	54.1 ±0.2	nd	3.38 ±0.02	nd	nd	nd	nd	nd	25.01	17.48	57.52	3.38	54.14	17.48
**Purple Wheat - New Bred Line**																				
DS8526-2	2.17 ±0.03	22.8 ±0.1	0.114 ±0.002	nd	5.28 ±0.15	21.5 ±0.2	44.7 ±0.3	nd	2.69 ±0.03	nd	0.611 ±0.004	nd	nd	nd	30.21	22.31	47.48	2.69	44.80	22.31
DS8526-2$_{\text{LUHS29}}$	nd	16.4 ±0.1	nd	nd	4.40 ±0.03	19.3 ±0.1	42.8 ±0.2	nd	7.45 ±0.05	nd	2.36 ±0.08	nd	nd	7.12 ±0.04	20.83	28.89	50.29	7.45	42.83	28.89
DS8526-2$_{\text{LUHS245}}$	1.19 ±0.02	20.3 ±0.2	0.315 ±0.006	nd	4.95 ±0.08	23.5 ±0.2	45.5 ±0.3	nd	2.80 ±0.02	nd	0.600 ±0.004	0.622 ±0.003	nd	nd	26.49	24.51	49.01	2.81	46.20	24.51
DS8526-2$_{\text{LUHS122}}$	nd	17.7 ±0.1	nd	nd	4.09 ±0.12	20.2 ±0.2	53.8 ±0.3	nd	3.28 ±0.07	nd	0.824 ±0.003	nd	nd	nd	21.76	21.06	57.18	3.29	53.89	21.06

LUHS29 – fermented with *Pediococcus acidilactici* LUHS29 strain; LUHS245 – fermented with *Lactobacillus uvarum* LUHS245strain; LUHS122 – fermented with *Lactobacillus plantarum* LUHS122 strain; C14:0 – methyl tetradecanoate; C16:0 – methyl palmitate; C16:1– methyl palmitoleate; C17:0 – methyl heptadecanoate; C18:0 – methyl stearate; C18:1 – cis-trans-9- oleic acid methyl ester; C18:2 – methyl linoleate; C18:3 γ – gamma- linolenic acid methyl ester C18:3 α – alfa linolenic acid methyl ester; C20:0 – eicosanoic acid methyl ester; C20:1 – cis-11-eicosenoic acid methyl ester; C20:4 – cis-5.8.11.14-eicosatetraenoic acid methyl ester; C22:0 – methyl docosanoate; C22:1 – cis-13-docosenoic acid methyl ester; SFA – saturated fatty acids, MUFA – monounsaturated fatty acids, PUFA – polyunsaturated fatty acids.

Data are represented as means (n = 3) ± SE. [a-c] Means with different letters in column are significantly different between the same variety non-treated and fermented samples ($p \leq 0.05$). [A-D] Means with different letters in column are significantly different between the different variety samples (between non-treated and between with the same LAB strain treated samples) ($p \leq 0.05$).

Table S8. The influence of the wheat variety and type of LAB on the fatty acids content in WW samples.

Source	Dependent Variable	p
Wheat variety	C14:0	0.0001
	C16:0	0.610
	C16:1	0.0001
	C17:0	0.0001
	C18:0	0.0001
	C18:1 cis-trans	0.201
	C18:2	0.757
	C18:3 γ	0.0001
	C18:3 α	0.064
	C20:0	0.0001
	C20:1	0.002
	C20:4	0.0001
	C22:0	0.004
	C22:1	0.0001
The type of LAB	C14:0	0.0001
	C16:0	0.118
	C16:1	0.0001
	C17:0	0.0001
	C18:0	0.0001
	C18:1 cis-trans	0.033
	C18:2	0.239
	C18:3 γ	0.0001
	C18:3 α	0.515
	C20:0	0.0001
	C20:1	0.0001
	C20:4	0.0001
	C22:0	0.004
	C22:1	0.0001
Wheat variety * The type of LAB	C14:0	0.0001
	C16:0	0.781
	C16:1	0.0001
	C17:0	0.0001
	C18:0	0.0001
	C18:1 cis-trans	0.305
	C18:2	0.193
	C18:3 γ	0.0001
	C18:3 α	0.569
	C20:0	0.0001
	C20:1	0.0001
	C20:4	0.0001
	C22:0	0.0001
	C22:1	0.0001

C14:0 – methyl tetradecanoate; C16:0 – methyl palmitate; C16:1– methyl palmitoleate; C17:0 – methyl heptadecanoate; C18:0 – methyl stearate; C18:1 – cis-trans-9- oleic acid methyl ester; C18:2 – methyl linoleate; C18:3 γ – gamma- linolenic acid methyl ester C18:3 α – alfa linolenic acid methyl ester; C20:0 – eicosanoic acid methyl ester; C20:1 – cis-11-eicosenoic acid methyl ester; C20:4 – cis-5.8.11.14-eicosatetraenoic acid methyl ester; C22:0 – methyl docosanoate; C22:1 – cis-13-docosenoic acid methyl ester.

The influence of analysed factors and their interaction is significant, when $p \leq 0.05$.
