# Supplementary material for: Comparison Study of Nontreated and Fermented Wheat Varieties ‘Ada’, ‘Sarta’, and New Breed Blue and Purple Wheat Lines Wholemeal Flour
Source: Biology (Basel). 2022 Jun 27;11(7):966. doi: 10.3390/biology11070966 (PMC9312326; doi:10.3390/biology11070966)
Supplement: Supplementary file 1 [file biology-11-00966-s001.zip › Supplementary File S5. Wheat samples volatile compounds.pdf]

Table S9. Volatile compounds, which in at least one wheat whole meal sample is >5%.

RT	Volatile compound	'Ada'	'Ada'$_{LUHS29}$	'Ada'$_{LUHS245}$	'Ada'$_{LUHS122}$	'Sarta'	'Sarta'$_{LUHS29}$	'Sarta'$_{LUHS245}$	'Sarta'$_{LUHS122}$	DS8472-5	DS8472-5$_{LUHS29}$	DS8472-5 $_{LUHS245}$	DS8472-5 $_{LUHS122}$	DS8526-2	DS8526-2$_{LUHS29}$	DS8526-2$_{LUHS245}$	DS8526-2 $_{LUHS122}$
2.34	Acetic acid	nd	31.8 ±2.8	11.7 ±0.7	6.34 ±0.41	nd	13.4 ±1.1	4.26 ±0.34	10.7 ±0.9	nd	23.3 ±2.2	nd	6.36 ±0.32	nd	11.3 ±0.6	nd	1.95±0.14
3.13	1-Butanol	nd	nd	nd	nd	nd	nd	nd	nd	nd	nd	nd	nd	nd	nd	nd	8.03±0.56
3.75	Acetoin	32.8 ±2.6	nd	nd	nd	5.21 ±0.36	nd	nd	nd	6.65 ±0.59	nd	nd	nd	nd	nd	nd	nd
4.99	1-Pentanol	27.7 ±1.4	2.00 ±0.11	nd	nd	nd	2.34 ±0.18	1.66 ±0.13	nd	nd	nd	nd	nd	nd	nd	nd	nd
5.50	2,3-Butanediol	22.1 ±2.1	nd	3.20 ±0.19	48.3 ±3.1	10.4 ±0.7	nd	nd	nd	53.8 ±4.8	nd	12.3 ±1.2	1.94 ±0.09	61.1 ±3.4	nd	38.4±2.5	0.739±0.051
5.75	Hexanal	nd	2.67 ±0.15	6.16 ±0.37	nd	29.9 ±2.1	6.66 ±0.50	12.7± 1.1	28.8 ±2.4	nd	nd	nd	nd	nd	5.21 ±0.31	nd	nd
5.83	Ethyl butyrate	nd	nd	nd	nd	nd	nd	nd	nd	nd	nd	nd	nd	nd	nd	nd	19.9±1.4
6.10	Ethyl lactate	nd	3.61 ±0.21	nd	nd	nd	5.05 ±0.38	nd	nd	nd	5.99 ±0.57	nd	nd	nd	nd	nd	nd
7.14	Butanoic acid	nd	nd	nd	nd	nd	nd	nd	nd	nd	nd	nd	nd	nd	nd	nd	37.4 ±2.6
7.57	1-Hexanol	5.80 ±0.29	6.70 ±0.37	5.43 ±0.33	3.27 ±0.21	20.5 ±1.44	10.0 ±0.8	4.75 ±0.38	2.30 ±0.20	9.89 ±0.89	7.78 ±0.74	10.13 ±1.01	0.321 ±0.016	6.55 ±0.36	4.66 ±0.28	4.30 ±0.28	nd
8.04	L-Lactic acid	nd	2.42 ±0.13	4.72 ±0.29	nd	nd	nd	0.956 ±0.076	nd	nd	nd	9.33 ±0.93	nd	nd	7.41 ±0.44	nd	nd
9.74	4-methylpentanoic acid	nd	nd	nd	nd	nd	nd	nd	nd	nd	nd	nd	54.8 ±4.7	nd	nd	nd	nd
9.87	2-Heptenal	0.175 ±0.009	1.13 ±0.06	3.89 ±0.23	0.616 ±0.041	nd	4.44 ±0.33	6.21 ±0.50	1.85 ±0.16	0.910 ±0.082	0.640 ±0.064	2.69 ±0.27	nd	nd	3.21 ±0.19	0.910 ±0.059	0.177 ±0.012
10.5	Hexanoic acid	nd	5.20 ±0.29	2.55 ±0.16	1.65 ±0.11	4.63 ±0.32	4.27 ±0.32	3.56 ±0.28	2.52 ±0.21	nd	9.07 ±0.86	5.04 ±0.50	0.753 ±0.038	nd	2.97 ±0.18	2.70 ±0.18	0.543 ±0.038
10.7	2-pentylfuran	0.780 ±0.041	4.53 ±0.25	3.97 ±0.24	2.07 ±0.13	2.63 ±0.18	4.38 ±0.34	5.34 ±0.43	4.52 ±0.38	1.26 ±0.11	4.76 ±0.45	4.74 ±0.47	nd	nd	4.52 ±0.27	2.24 ±0.15	0.250 ±0.018
10.8	Butylbutanoate	nd	nd	nd	nd	nd	nd	nd	nd	nd	nd	nd	nd	nd	nd	nd	17.9 ±1.3
11.0	Hexanoic acid ethyl ester	nd	2.56 ±0.14	nd	1.62 ±0.11	nd	2.46 ±0.18	nd	3.05 ±0.26	nd	5.07 ±0.48	3.45 ±0.35	22.4 ±1.1	nd	1.80 ±0.11	3.00 ±0.20	nd
11.8	3-ethyl-2-methyl-1,3-hexadiene	nd	3.92 ±0.22	9.48 ±0.57	4.76 ±0.31	0.928 ±0.065	6.26 ±0.47	8.34 ±0.67	3.99 ±0.34	nd	3.53 ±0.34	9.83 ±0.98	1.58 ±0.08	0.725 ±0.040	7.58 ±0.45	5.87 ±0.38	0.768 ±0.054
12.1	Benzeneacetaldehyde	nd	3.55 ±0.21	3.21 ±0.18	3.70 ±0.25	nd	2.78 ±0.21	2.43 ±0.19	2.70 ±0.23	nd	3.22 ±0.31	2.51 ±0.25	1.18 ±0.06	nd	3.35 ±0.20	3.14 ±0.20	0.669 ±0.047
12.4	Oct-(2E)-enal	1.03 ±0.05	4.90 ±0.27	12.6 ±0.77	5.43 ±0.35	4.19 ±0.29	8.24 ±0.67	14.1 ±1.1	8.97 ±0.76	1.61 ±0.14	nd	6.37 ±0.64	0.664 ±0.033	1.26 ±0.07	11.4 ±0.7	5.96 ±0.39	2.00 ±0.14
17.6	4-ethylguaiacol	nd	0.123 ±0.007	nd	0.512 ±0.033	nd	nd	0.356 ±0.031	nd	nd	nd	1.02 ±0.10	0.216± 0.011	1.55 ±0.09	1.61 ±0.10	8.27 ±0.54	1.40 ±0.09
18.3	Deca-(2E,4E)-dien-1-ol	nd	nd	nd	nd	nd	3.39 ±0.25	6.33 ±0.51	6.10 ±0.52	0.345 ±0.031	nd	nd	nd	nd	nd	nd	nd
18.3	4-vinylguaiacol	nd	1.30 ±0.07	5.51 ±0.33	7.69 ±0.49	5.91 ±0.41	nd	nd	nd	nd	3.06 ±0.29	6.66 ±0.66	1.67 ±0.08	16.2 ±0.89	6.10 ±0.37	8.28 ±0.57	nd

LUHS29 – fermented with *Pediococcus acidilactici* LUHS29 strain; LUHS245 – fermented with *Lactobacillus uvarum* LUHS245strain; LUHS122 – fermented with *Lactobacillus plantarum* LUHS122 strain. Data are represented as means (n = 3)) ± SE.

Table S10. Volatile compounds, which in at least one wheat whole meal sample is >1 and <5%.

RT	Volatile compound	'Ada'	'Ada' LUHS29	'Ada' LUHS245	'Ada' LUHS122	'Sarta'	'Sarta' LUHS29	'Sarta' LUHS245	'Sarta' LUHS122	DS8472-5	DS8472-5 LUHS29	DS8472-5 LUHS245	DS8472-5 LUHS122	DS8526-2	DS8526-2 LUHS29	DS8526-2 LUHS245	DS8526-2 LUHS122
8.71	2,5-dimethylpyrazine	nd	nd	0.246 ±0.022	nd	nd	nd	nd	nd	nd	1.27 ±0.08	nd	0.434 ±0.043	nd	0.459 ±0.037	nd	nd
9.97	Benzaldehyde	nd	nd	0.680 ±0.061	0.506 ±0.043	nd	nd	0.848 ±0.059	1.42 ±0.09	nd	nd	nd	nd	nd	3.10 ±0.25	nd	0.171 ±0.010
10.2	1-Heptanol	0.551 ±0.055	0.825 ±0.078	nd	nd	1.03 ±0.08	0.763 ±0.057	nd	nd	0.549 ±0.036	1.83 ±0.11	0.766 ±0.042	nd	0.718 ±0.065	1.37 ±0.11	nd	nd
10.4	1-Octen-3-ol	0.533 ±0.053	3.07 ±0.29	3.40 ±0.31	nd	nd	3.47 ±0.26	1.52 ±0.11	2.10 ±0.14	0.445 ±0.029	5.68 ±0.34	3.09 ±0.17	nd	0.511 ±0.046	3.74 ±0.30	nd	nd
12.3	4-methylhexanoic acid	nd	nd	nd	nd	nd	nd	nd	nd	nd	nd	nd	1.44 ±0.14	nd	nd	nd	nd
12.8	Heptanoic acid	nd	0.358 ±0.034	0.324 ±0.031	nd	nd	0.386 ±0.029	0.209 ±0.014	0.202 ±0.013	nd	1.52 ±0.09	0.522 ±0.029	0.171 ±0.017	nd	0.919 ±0.073	0.520 ±0.036	0.024 ±0.001
13.3	2-Nonanone	0.979 ±0.098	0.787 ±0.074	1.90 ±0.17	0.410 ±0.034	nd	nd	nd	nd	0.381 ±0.025	0.573 ±0.034	1.26 ±0.07	nd	nd	nd	0.767 ±0.053	nd
13.6	Nonanal	nd	0.791 ±0.075	0.790 ±0.071	0.465 ±0.040	0.422 ±0.034	0.629 ±0.40	0.860 ±0.060	0.631 ±0.041	1.50 ±0.10	0.217 ±0.013	0.342 ±0.019	0.103 ±0.011	nd	0.647 ±0.051	0.412 ±0.029	0.080 ±0.005
13.8	Phenethyl alcohol	0.499 ±0.049	0.979 ±0.093	0.984 ±0.089	0.818 ±0.070	nd	0.274 ±0.021	nd	0.349 ±0.022	nd	1.14 ±0.07	1.09 ±0.06	0.265 ±0.027	0.594 ±0.053	0.830 ±0.066	0.445 ±0.031	0.093 ±0.006
14.9	(E)-2-Nonenal	0.346 ±0.004	0.615 ±0.058	0.987 ±0.088	1.24 ±0.11	2.33 ±0.19	1.07 ±0.08	1.45 ±0.10	2.75 ±0.179	0.883 ±0.057	0.939 ±0.056	0.873 ±0.048	0.531 ±0.053	1.01 ±0.09	1.14 ±0.09	1.51 ±0.11	0.476 ±0.029
15.0	4-ethylphenol	nd	0.218 ±0.021	0.253 ±0.022	nd	nd	0.374 ±0.028	0.522 ±0.037	nd	nd	0.939 ±0.055	1.14 ±0.06	0.091 ±0.090	nd	1.09 ±0.08	nd	0.022 ±0.001
15.1	Octanoic acid	1.01 ±0.11	2.15 ±0.20	2.68 ±0.24	1.44 ±0.12	2.02 ±0.16	3.11 ±0.23	3.20 ±0.22	1.86 ±0.12	0.892 ±0.058	5.23 ±0.31	2.26 ±0.12	0.651 ±0.049	1.24 ±0.11	3.00 ±0.24	1.52 ±0.10	0.518 ±0.031
15.7	Ethyl octanoate	0.112 ±0.011	0.819 ±0.078	0.868 ±0.078	1.14 ±0.09	0.377 ±0.030	0.825 ±0.062	1.12 ±0.08	2.72 ±0.18	1.10 ±0.07	1.37 ±0.08	0.876 ±0.048	0.329 ±0.031	0.218 ±0.019	0.279 ±0.022	1.98 ±0.14	0.345 ±0.021
16.1	(E,E)-2,4-Nonadienal	0.269 ±0.027	0.257 ±0.02	0.481 ±0.043	0.156 ±	0.571 ±0.046	0.598 ±0.045	1.39 ±0.10	0.966 ±0.063	0.872 ±0.060	0.177 ±0.011	0.399 ±0.021	0.227 ±0.026	nd	0.321 ±0.026	0.339 ±0.02	0.178 ±0.011
17.2	Nonanoic acid	1.62 ±0.16	4.98 ±0.47	1.86 ±0.17	1.37 ±0.17	3.25 ±0.26	3.28 ±0.27	1.44 ±0.11	0.214 ±0.014	1.51 ±0.09	4.33 ±0.26	2.28 ±0.13	0.262 ±0.060	2.69 ±0.24	2.12 ±0.17	1.63 ±0.11	0.170 ±0.010
17.9	(E,E)-2,4-Decadienal	nd	1.03 ±0.09	3.58 ±0.32	1.21 ±0.10	1.73 ±0.14	1.43 ±0.11	3.89 ±0.27	2.43 ±0.16	3.54 ±0.23	0.931 ±0.056	nd	0.237 ±0.024	1.25 ±0.11	1.92 ±0.15	2.26 ±0.16	1.11 ±0.07
18.3	4-methyl-1-(pent-4-en-1-yl)-2,3-Diazabicyclo[2.2.1] hept-2-ene	0.520 ±0.052	nd	nd	nd	nd	nd	nd	nd	8.07 ±0.52	nd	nd	nd	nd	nd	nd	2.58 ±0.15
18.4	4-ethyl-3-Nonen-5-yne	nd	0.659 ±0.062	2.26 ±0.20	0.846 ±0.071	nd	2.72 ±0.20	3.53 ±0.24	1.32 ±0.09	nd	0.406 ±0.024	3.03 ±0.17	0.209 ±0.022	nd	3.65 ±0.29	0.872 ±0.061	0.300 ±0.018
19.0	Ethylhydrocinnamate	nd	nd	nd	nd	nd	nd	nd	nd	nd	nd	nd	1.06 ±0.11	nd	nd	nd	nd
19.4	dihydro-5-pentyl-2(3H)-Furanone	0.255 ±0.025	0.536 ±0.051	0.605 ±0.054	0.398 ±0.033	1.59 ±0.13	1.23 ±0.09	0.945 ±0.066	0.865 ±0.057	0.528 ±0.034	1.05 ±0.06	0.931 ±0.051	0.223 ±0.021	0.843 ±0.076	0.890 ±0.071	0.591 ±0.041	0.154 ±0.009
19.7	trans-4,5-Epoxy-(E)-2-decenal	nd	0.388 ±0.037	1.64 ±0.15	0.366 ±0.031	nd	0.988 ±0.074	2.56 ±0.18	0.956 ±0.062	nd	nd	0.570 ±0.031	0.081 ±0.009	0.214 ±0.019	0.847 ±0.067	0.418 ±0.029	0.237 ±0.014
23.0	Dodecanoic acid	nd	nd	nd	nd	nd	nd	nd	nd	0.113 ±0.007	2.06 ±0.12	0.543 ±0.029	0.046 ±0.005	0.115 ±0.010	0.263 ±0.021	0.728 ±0.050	0.028 ±0.002

LUHS29 – fermented with *Pediococcus acidilactici* LUHS29 strain; LUHS245 – fermented with *Lactobacillus uvarum* LUHS245strain; LUHS122 – fermented with *Lactobacillus plantarum* LUHS122 strain. Data are represented as means (n = 3)) ± SE.

Table S11. Volatile compounds, which in at least one wheat whole meal sample is <1%.

RT	Volatile compound	‘Ada’	‘Ada’$_{LUHS29}$	‘Ada’$_{LUHS245}$	‘Ada’$_{LUHS122}$	‘Sarta’	‘Sarta’$_{LUHS29}$	‘Sarta’$_{LUHS245}$	‘Sarta’$_{LUHS122}$	DS8472-5	DS8472-5$_{LUHS29}$	DS8472-5$_{LUHS245}$	DS8472-5$_{LUHS122}$	DS8526-2	DS8526-2$_{LUHS29}$	DS8526-2$_{LUHS245}$	DS8526-2$_{LUHS122}$
7.45	3-methylbutanoic acid	nd	nd	nd	nd	nd	nd	nd	nd	nd	nd	nd	nd	nd	nd	nd	0.033 ±0.002
7.60	2-methylbutanoic acid	nd	nd	nd	nd	nd	nd	nd	nd	nd	nd	nd	nd	nd	nd	nd	0.033 ±0.002
8.18	Pentanoic acid	nd	1.04 ±0.07	0.611 ±0.031	nd	nd	nd	nd	nd	nd	nd	nd	nd	nd	0.657 ±0.057	nd	nd
8.40	Heptanal	nd	nd	nd	nd	0.927 ±0.079	nd	nd	nd	nd	nd	nd	nd	nd	nd	nd	nd
11.2	(E,E)-2,4-heptadienal	nd	nd	nd	nd	nd	0.255 ±0.019	0.253 ±0.016	0.175 ±0.167	nd	nd	nd	nd	nd	nd	nd	0.104 ±0.007
11.2	Hexyl acetate	nd	nd	nd	nd	nd	0.705 ±0.053	0.176 ±0.011	0.216 ±0.021	nd	nd	nd	nd	nd	nd	nd	nd
11.3	Acetic acid hexyl ester	nd	0.426 ±0.032	nd	0.098 ±0.009	nd	nd	nd	nd	nd	0.351 ±0.028	nd	nd	nd	nd	nd	nd
11.9	Oct-3-en-2-one	nd	nd	nd	nd	nd	nd	nd	0.413 ±0.039	nd	nd	nd	nd	nd	nd	nd	nd
12.7	(E)-2-Octen-1-ol	0.170 ±0.014	nd	nd	nd	0.506 ±0.043	0.554 ±0.042	0.365 ±0.024	0.378 ±0.036	0.353 ±0.032	0.574 ±0.046	0.578 ±0.041	0.192 ±0.012	0.478 ±0.045	nd	0.448 ±0.033	nd
12.7	1-Octanol	0.346 ±0.028	0.704 ±0.049	1.12 ±0.06	0.727 ±0.065	0.363 ±0.031	1.03 ±0.08	0.755 ±0.049	nd	0.608 ±0.055	nd	0.638 ±0.044	nd	0.551 ±0.052	nd	nd	nd
13.3	2-butyltetrahydrofuran	nd	nd	nd	nd	nd	0.241 ±0.018	1.14 ±0.07	1.45 ±0.14	nd	nd	nd	0.277 ±0.017	nd	0.228 ±0.019	nd	0.205 ±0.014
13.4	Heptanoic acid ethyl ester	nd	nd	nd	0.188 ±0.017	nd	nd	nd	nd	nd	0.184 ±0.015	nd	0.141 ±0.008	nd	nd	0.120 ±0.009	nd
13.4	(Z)-2-Nonen-4-yn-1-ol	nd	nd	nd	nd	nd	nd	nd	0.804 ±0.076	nd	nd	nd	nd	nd	nd	nd	nd
13.5	Undecane	nd	nd	nd	nd	nd	nd	nd	nd	0.242 ±0.022	nd	nd	nd	nd	nd	nd	nd
14.4	Butanoic acid pentyl ester	nd	nd	nd	nd	nd	nd	nd	0.406 ±0.038	nd	nd	nd	nd	nd	nd	nd	0.082 ±0.006
14.5	5,5,6-Trimethylhept-3-en-2-one	nd	nd	nd	nd	nd	0.183 ±0.014	0.122 ±0.008	0.175 ±0.017	0.185 ±0.017	0.077 ±0.006	nd	nd	nd	nd	nd	0.033 ±0.002
15.2	Benzoic acid ethyl ester	0.418 ±0.033	nd	nd	0.338 ±0.031	nd	nd	nd	nd	0.925 ±0.083	nd	nd	0.130 ±0.008	0.659 ±0.062	nd	0.735 ±0.054	nd
15.3	Butanedioic acid diethyl ester	nd	nd	nd	0.510 ±0.046	nd	nd	nd	nd	nd	nd	nd	nd	nd	nd	0.449 ±0.033	nd
15.5	Hexylbutyrate	nd	nd	nd	nd	nd	nd	nd	0.129 ±0.012	nd	nd	nd	nd	nd	nd	nd	nd
15.8	Dodecane	0.299 ±0.024	0.272 ±0.019	nd	nd	nd	nd	nd	nd	0.432 ±0.039	0.212 ±0.017	0.157 ±0.011	0.041 ±0.002	0.172 ±0.016	nd	0.098 ±0.007	0.058 ±0.004
15.9	Decanal	nd	0.240 ±0.017	0.272 ±0.014	0.116 ±0.011	nd	0.357 ±0.027	0.330 ±0.021	0.133 ±0.013	0.138 ±0.012	nd	0.444 ±0.031	0.077 ±0.005	nd	nd	nd	nd
16.0	2-(ethylthio)propanoic acid ethyl ester	nd	nd	nd	nd	nd	nd	nd	nd	nd	nd	nd	0.088 ±0.005	nd	nd	nd	nd
16.2	Sorbic acid vinyl ester	nd	nd	nd	nd	nd	nd	nd	nd	nd	nd	nd	0.115 ±0.007	nd	nd	nd	nd
16.5	Benzothiazole	nd	nd	nd	nd	nd	nd	nd	nd	nd	nd	nd	0.036 ±0.002	nd	nd	nd	nd
16.8	Benzeneacetic acid ethyl ester	nd	nd	nd	nd	nd	nd	nd	nd	nd	nd	0.145 ±0.012	0.049 ±0.003	nd	nd	0.149 ±0.011	nd
17.0	4a,5-Dimethylhexahydro-	nd	0.506 ±0.035	0.347 ±0.017	0.147 ±0.013	nd	0.389 ±0.029	0.278 ±0.018	0.062 ±0.006	nd	nd	0.240 ±0.011	nd	nd	nd	nd	nd

	4H-1,3-benzodioxin-4-one																
17.2	Dec-(2E)-enal	nd	nd	0.133 ±0.007	nd	nd	0.206 ±0.015	0.631 ±0.041	0.109 ±0.011	0.330 ±0.029	nd	nd	nd	nd	nd	nd	0.033 ±0.002
17.6	4,6-dimethyldodecane	0.871 ±0.069	nd	nd	nd	nd	nd	nd	0.179 ±0.017	0.379 ±0.034	0.485 ±0.039	nd	nd	nd	nd	nd	nd
17.8	2-Undecanone	nd	nd	nd	nd	nd	nd	nd	nd	nd	nd	2.30 ±0.16	nd	nd	nd	nd	nd
17.9	Benzo-2,3-pyrrole	nd	nd	nd	0.805 ±0.072	nd	nd	nd	nd	nd	nd	nd	0.094 ±0.006	nd	nd	nd	nd
18.0	Tridecane	0.150 ±0.012	0.173 ±0.012	nd	nd	nd	0.114 ±0.009	0.085 ±0.006	0.091 ±0.009	0.304 ±0.027	0.198 ±0.016	0.149 ±0.011	nd	0.164 ±0.016	0.173 ±0.015	0.119 ±0.009	0.033 ±0.002
19.3	n-Decanoic acid	0.233 ±0.019	1.05 ±0.07	0.380 ±0.019	0.259 ±0.023	0.495 ±0.042	0.629 ±0.047	0.208 ±0.014	nd	0.069 ±0.006	0.581 ±0.046	0.115 ±0.008	nd	0.191 ±0.018	0.163 ±0.014	0.264 ±0.021	nd
19.6	cis-4,5-Epoxy-(E)-2-decenal	nd	0.175 ±0.012	0.658 ±0.033	0.121 ±0.011	nd	0.364 ±0.027	0.964 ±0.063	0.338 ±0.032	nd	nd	0.111 ±0.007	nd	nd	0.297 ±0.026	0.092 ±0.007	0.069 ±0.005
19.812	Butyl caprylate	nd	nd	nd	nd	nd	nd	nd	nd	nd	nd	nd	nd	nd	nd	nd	0.136 ±0.009
19.9	Ethyl decanoate	nd	nd	nd	0.095 ±0.009	nd	nd	0.044 ±0.003	0.339 ±0.031	nd	nd	nd	nd	nd	nd	nd	nd
20.0	Tetradecane	0.361 ±0.028	0.431 ±0.031	0.345 ±0.017	0.281 ±0.025	nd	nd	nd	0.238 ±0.023	0.655 ±0.059	0.555 ±0.044	0.468 ±0.033	0.095 ±0.009	0.333 ±0.032	0.351 ±0.031	0.259 ±0.019	0.303 ±0.021
20.3	2,4,7,9-Tetramethyl-5-decyn-4,7-diol	nd	nd	nd	nd	nd	nd	nd	nd	0.250 ±0.023	0.301 ±0.024	0.275 ±0.019	nd	0.155 ±0.015	nd	0.146 ±0.011	nd
21.2	2,6,10-trimethyldodecane,	nd	nd	nd	nd	nd	nd	nd	nd	0.149 ±0.013	nd	0.095 ±0.007	0.031 ±0.003	nd	0.143 ±0.012	nd	0.018 ±0.001
22.2	2,4-bis(1,1-dimethylethyl)phenol	nd	nd	nd	nd	nd	nd	nd	nd	nd	0.150 ±0.012	0.092 ±0.006	nd	nd	nd	nd	nd
22.7	Pentadecane	nd	nd	nd	nd	nd	nd	nd	nd	0.080 ±0.007	0.161 ±0.013	0.099 ±0.007	0.029 ±0.003	0.177 ±0.017	0.117 ±0.011	nd	nd
23.4	(Z)-3-Hexadecene	nd	nd	nd	nd	nd	nd	nd	nd	nd	nd	nd	nd	nd	nd	nd	0.077 ±0.005
23.7	Hexadecane	nd	nd	nd	nd	nd	nd	nd	nd	nd	nd	nd	nd	0.185 ±0.018	nd	nd	0.430 ±0.029
23.8	Propanoic acid, 2-methyl-, 1-(1,1-dimethylethyl)-2-methyl-1,3-propanediyl ester	nd	nd	nd	nd	nd	nd	nd	nd	nd	nd	nd	nd	nd	nd	nd	0.013 ±0.001

Volatile compounds, which in at least in one wheat wholemeal sample are >5% (LUHS29 – fermented with *Pediococcus acidilactici* LUHS29 strain; LUHS245 – fermented with *Lactobacillus uvarum* LUHS245strain; LUHS122 – fermented with *Lactobacillus plantarum* LUHS122 strain. Data are represented as means (n = 3)).

Table S12. The influence of the wheat variety and type of LAB on the acidity, chromaticity, and microbiological characteristics of the WW samples.

Dependent Variable	Significance (p) of the wheat variety	Significance (p) of the type of LAB	Significance of the wheat variety * The type of LAB interaction
Acetic acid	0.0001	0.0001	0.0001
1-Butanol	0.0001	0.0001	0.0001
Acetoin	0.0001	0.0001	0.0001
1-Pentanol	0.0001	0.0001	0.0001
2,3-Butanediol	0.0001	0.0001	0.0001
Hexanal	0.0001	0.0001	0.0001
Ethyl butyrate	0.0001	0.0001	0.0001
Ethyl lactate	0.0001	0.0001	0.0001
Butanoic acid	0.0001	0.0001	0.0001
1-Hexanol	0.132	0.003	0.026
L-Lactic acid	0.998	0.0001	0.0001
4-methylpentanoic acid	0.0001	0.0001	0.0001
2-Heptenal	0.797	0.012	0.0001
Hexanoic acid	0.0001	0.0001	0.0001
2-pentylfuran	0.004	0.0001	0.007
Butylbutanoate	0.0001	0.0001	0.0001
Hexanoic acid ethyl ester	0.0001	0.0001	0.0001
3-ethyl-2-methyl-1,3-hexadiene	0.0001	0.0001	0.0001
Benzeneacetaldehyde	0.0001	0.0001	0.0001
Oct-(2E)-enal	0.005	0.0001	0.0001
4-ethylguaiacol	0.0001	0.0001	0.0001
Deca-(2E,4E)-dien-1-ol	0.0001	0.139	0.0001
4-vinylguaiacol	0.003	0.074	0.003
2,5-dimethylpyrazine	0.0001	0.0001	0.0001
Benzaldehyde	0.0001	0.0001	0.0001
1-Heptanol	0.0001	0.0001	0.0001
1-Octen-3-ol	0.003	0.0001	0.0001
4-methylhexanoic acid	0.023	0.030	0.010
Heptanoic acid	0.031	0.0001	0.003
2-Nonanone	0.0001	0.0001	0.0001
Nonanal	0.0001	0.0001	0.0001
Phenethyl alcohol	0.0001	0.002	0.0001
(E)-2-Nonenal	0.022	0.070	0.0001
4-ethylphenol	0.0001	0.0001	0.0001
Octanoic acid	0.0001	0.069	0.010
Ethyl octanoate	0.941	0.006	0.009
(E,E)-2,4-Nonadienal	0.0001	0.0001	0.0001
Nonanoic acid	0.001	0.0001	0.0001
(E,E)-2,4-Decadienal	0.0001	0.0001	0.0001

4-methyl-1-(pent-4-en-1-yl)-2,3-Diazabicyclo[2.2.1] hept-2-ene	0.0001	0.0001	0.0001
4-ethyl-3-Nonen-5-yne	0.0001	0.0001	0.0001
Ethylhydrocinnamate	0.0001	0.002	0.0001
dihydro-5-pentyl-2(3H)-Furanone	0.171	0.005	0.0001
trans-4,5-Epoxy-(E)-2-decenal	0.0001	0.0001	0.0001
Dodecanoic acid	0.0001	0.0001	0.0001
3-methylbutanoic acid	0.0001	0.0001	0.0001
2-methylbutanoic acid	0.0001	0.0001	0.0001
Pentanoic acid	0.029	0.028	0.001
Heptanal	0.0001	0.0001	0.0001
(E,E)-2,4-heptadienal	0.0001	0.001	0.0001
Hexyl acetate	0.0001	0.0001	0.0001
Acetic acid hexyl ester	0.0001	0.0001	0.0001
Oct-3-en-2-one	0.0001	0.0001	0.0001
(E)-2-Octen-1-ol	0.0001	0.0001	0.0001
1-Octanol	0.0001	0.0001	0.0001
2-butyltetrahydrofuran	0.0001	0.0001	0.0001
Heptanoic acid ethyl ester	0.0001	0.0001	0.0001
(Z)-2-Nonen-4-yn-1-ol	0.005	0.010	0.0001
Undecane	0.011	0.008	0.001
Butanoic acid pentyl ester	0.0001	0.0001	0.0001
5,5,6-Trimethylhept-3-en-2-one	0.0001	0.0001	0.0001
Benzoic acid ethyl ester	0.0001	0.0001	0.0001
Butanedioic acid diethyl ester	0.0001	0.0001	0.0001
Hexylbutyrate	0.0001	0.0001	0.0001
Dodecane	0.0001	0.0001	0.0001
Decanal	0.0001	0.0001	0.0001
2-(ethylthio)propanoic acid ethyl ester	0.0001	0.0001	0.0001
Sorbic acid vinyl ester	0.0001	0.0001	0.0001
Benzothiazole	0.0001	0.0001	0.0001
Benzeneacetic acid ethyl ester	0.0001	0.0001	0.0001
4a,5-Dimethylhexahydro-4H-1,3-benzodioxin-4-one	0.0001	0.0001	0.001
Dec-(2E)-enal	0.001	0.080	0.001
4,6-dimethyldodecane	0.0001	0.000	0.0001
2-Undecanone	0.010	0.007	0.0001
Benzo-2,3-pyrrole	0.0001	0.0001	0.0001
Tridecane	0.0001	0.0001	0.0001
n-Decanoic acid	0.0001	0.0001	0.0001
cis-4,5-Epoxy-(E)-2-decenal	0.0001	0.0001	0.0001
Butyl caprylate	0.0001	0.0001	0.0001
Ethyl decanoate	0.0001	0.0001	0.0001
Tetradecane	0.0001	0.050	0.0001

2,4,7,9-Tetramethyl-5-decyn-4,7-diol	0.0001	0.0001	0.0001
2,6,10-trimethyldodecane,	0.0001	0.0001	0.0001
2,4-bis(1,1-dimethylethyl)phenol	0.0001	0.004	0.0001
Pentadecane	0.0001	0.0001	0.0001
(Z)-3-Hexadecene	0.0001	0.0001	0.0001
Hexadecane	0.0001	0.010	0.001
Propanoic acid, 2-methyl-, 1-(1,1-dimethylethyl)-2-methyl-1,3-propanediyl ester	0.0001	0.0001	0.0001

LAB – lactic acid bacteria. The influence of analysed factors and their interaction is significant, when $p \leq 0.05$. In blue colour marked values are not significant.
